# Exploring intermolecular contacts in multi-substituted benzaldehyde derivatives: X-ray, Hirshfeld surface and lattice energy analyses†

**DOI:** 10.1039/c9ra10752e

**Published:** 2020-04-29

**Authors:** Siya T. Hulushe, Meloddy H. Manyeruke, Marcel Louzada, Sergei Rigin, Eric C. Hosten, Gareth M. Watkins

**Affiliations:** Department of Chemistry, Rhodes University P.O. Box 94 Grahamstown 6139 South Africa g11h7156@campus.ru.ac.za; Department of Chemistry, New Mexico Highlands University Las Vegas New Mexico 87701 USA; Department of Chemistry, Nelson Mandela University P.O. Box 77000 Port Elizabeth 6031 South Africa

## Abstract

Crystal structures of six benzaldehyde derivatives (1–6) have been determined and their supramolecular networks were established by an X-ray crystallographic study. The study has shown that the compounds are linked by various intermolecular interactions such as weak C–H⋯O hydrogen bonding, and C–H⋯π, π–π and halogen bonding interactions which consolidate and strengthen the formation of these molecular assemblies. The carbonyl group generates diverse synthons in 1–6*via* intermolecular C–H⋯O hydrogen bonds. An interplay of C–H⋯O hydrogen bonds, and C–H⋯π and π–π stacking interactions facilitates the formation of multi-dimensional supramolecular networks. Crystal packings in 4 and 5 are further generated by type I halogen⋯halogen bonding interactions. The differences in crystal packing are represented by variation of substitution positions in the compounds. Structure 3 is isomorphous with 4 but there are subtle differences in their crystal packing. The nature of intermolecular contacts in the structures has been studied through the Hirshfeld surfaces and two-dimensional fingerprint plots which serve as a comparison in constructing different supramolecular networks. The intermolecular interaction energies are quantified utilizing theorectical calculations for the title compounds and various analogous structures retrieved from the Cambridge Structural Database (CSD). Also intermolecular interactions for the molecular pairs are exctrated from respective crystal structures. Essentially, there are some invariant and variable intermolecular contacts realized between different groups in all six structures. The *ab initio* DFT total lattice energy (*E*_Tot_) calculations showed a direct correlation with thermal strengths of the title compounds.

## Abstract 2

We report here crystal packing, and Hirshfeld surface and Lattice energy analyses of six new multi-substituted benzaldehyde derivatives for the first time.

## Graphical abstract

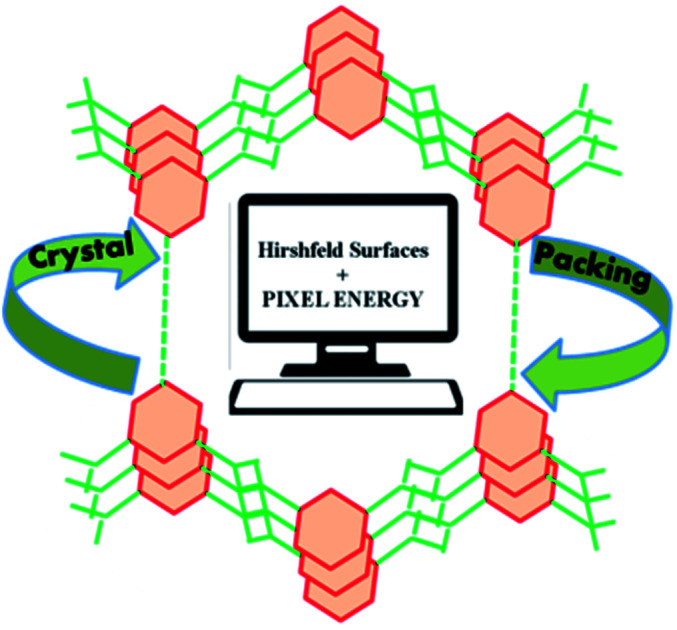

## 1. Introduction

Crystal engineering of supramolecular networks linked *via* intermolecular contacts continues to be a dynamic topic in the solid-state studies of self-assembly.^1^ Hydrogen bonds are recognized for their contribution in self-assembly when extended structures are constructed from synthons with aromatic moieties.^2^ Electronegative atoms such as O and N are recognized to form strong hydrogen bonds D–H⋯A (D = donor, A = acceptor) with an estimated interaction energy between 16–50 kJ mol^−1^.^2^ Intermolecular contacts or weak interactions (≤15 kJ mol^−1^) such as C–H⋯π, hydrogen/halogen bonds and π–π stacking are well-known to significantly influence the molecular assembly in organic compounds.^3^ Numerous synthons that incorporate intermolecular contacts such hydrogen/halogen bonds, C–H⋯π, lone pair–π and π–π stacking interactions significantly affect robustness to produce molecular solids with promising properties.^4^ Halogen bonding interactions established in many halogen-containing organic crystals are believed to enhance crystal stability.^5^ Essentially, these intermolecular contacts are said to be independently weaker and geometrically less well-defined, but their combined effect can be equally important as strong interactions.^6^ It is useful to study the diversity of hydrogen-bonding systems in molecules that contain a rigid benzyloxy core with different positions of substituents, and to explore their structural features including interplay of intermolecular contacts in building the possible supramolecular networks.^7^ It is of interest to explore the role of these intermolecular contacts in the molecular assembly of halogen-substituted (benzyloxy)benzaldehydes. An additional interest in the (benzyloxy)benzaldehyde moiety lies in its anticancer activity against HL-60 cells^8^ and also serves as important precursors in the design of new inhibitors of HIV-1 integrase.^9^ A search of the Cambridge Structural Database (version 5.40, November CSD 2018 release)^10^ for benzyloxybenzene derivatives (search + restricted to the aldehyde class) among the organic compounds returned 71 hits (excluding duplicate structures). Further restriction to (benzyloxy)benzaldehyde/(benzyloxy)benzoic acid derivatives (excluding solvates and cocrystals), only 15 structures^11^ with refcodes COBNUC, CUNMAZ, DUTRIU, DUTRIU01, DUTRIU02, EROHUP, KERDUH, IPEXEH, LELQUQ, LELRAX, MEQLIE, POMLUA, VOQFIS, XEVROF and XIMPAL are found. Compound 1 crystallizes in a new crystal form in space group *P*2_1_2_1_2_1_ which is different from the orthorhombic *Pna*2_1_ space group reported previously (DUTRIU, DUTRIU01 and DUTRIU02). Sze *et al.*, 2011 reported a similar structure to 6 (refcode: IPEXEH with space group *P*1̄) with different unit cell dimensions. Self-assembly mediated by weak interactions has been established as a convenient and prevailing protocol for the construction of geometrically well-defined structures. A suitable approach to deal with crystal structure prediction is represented by Hirshfeld surface^12^ based tool and this method provides a simplistic way of managing valuable statistics on trends in crystal packing. The variations in Hirshfeld surface and the analysis of the resultant 2D fingerprint plot^12^ offer a powerful means of quantifying the interactions within the crystal structures, drawing attention to significant similarity and differences between structures by individuating the packing motifs. In this paper, we report the synthesis of a series of multi-substituted benzaldehyde derivatives. Their structures were confirmed by spectroscopic methods and X-ray crystallography. Essentially, X-ray crystallographic analysis of the structures demonstrates the presence of multiple weak and non-covalent C–H⋯O hydrogen bonding, C–H⋯π, π–π stacking and halogen⋯halogen bonding interactions are likely to play an important role in the supramolecular ensembles of these non-planar layered benzaldehyde derivatives in the solid state. To investigate the effect of substituent positions on the resulting compounds as well as the molecular packing, we have examined six benzaldehydes (1–6) with different substituent positions in the aldehyde skeleton. In this paper, we have demonstrated that weak intermolecular contacts are stronger for benzyloxybenzaldehydes than their analogous structures reported by Chattopadhyay.^4^ Also, we have shown that there are subtle differences in crystal packing between these structures. In addition, a study of close intermolecular contacts in the title derivatives by Hirshfeld surface and lattice energy analyses is presented.

## 2. Material and methods

### 2.1 Materials

All commercially available chemicals and reagents were purchased from Sigma-Aldrich (Pty) Ltd and Merck (Pty) Ltd, and were used without further purification unless stated otherwise. Fourier-transform infrared (FT-IR) spectra were collected on PerkinElmer Spectrum 100 spectrometer with an ATR attachment. Mid-infrared (4000–650 cm^−1^) spectra were obtained by placing samples on a ZnSe crystal plate. The ^1^H and ^13^C NMR spectra were recorded on either a Bruker Fourier 300 or a 400 MHz spectrometer. Spectra were recorded in deuterated solvent CDCl_3_. All chemical shift values are reported in parts per million (ppm) referenced to residual solvent resonances (CDCl_3_*δ*_H_ 7.26, *δ*_C_ 77.2).

### 2.2 Synthesis and crystallization

Compound 1: a mixture of 4-hydroxybenzaldehyde (1.22 g, 10 mmol) and benzyl bromide (1.18 mL, 10 mmol) was refluxed in 10% aqueous sodium hydroxide (NaOH, 10 mL) solution for thirty minutes and then cooled to room temperature. Water was then added and the solid which formed was filtered, washed with water, dried and finally crystallised from hexane/water mixture affording 4-(benzyloxy) benzaldehyde as an off-white block crystals (0.8 g, 38%) mp 99–100 °C (lit.^8^ 99–100 °C); *ν*_max_/cm^−1^ 1213 (C–O), 1736 (C=O); *δ*_H_ (400 MHz; CDCl_3_) 5.15 (2H, s, PhCH_2_), 7.08 (2H, d, *J* = 8.7 Hz, ArH), 7.34–7.47 (5H, m, ArH overlapping), 7.84 (2H, d, *J* = 8.8 Hz, ArH) and 9.89 (1H, s, HC=O); *δ*_C_ (100 MHz; CDCl_3_) 70.4 (PhCH_2_), 115.2, 127.6, 128.4, 128.8, 130.2, 132.1, 136.0, 163.8 (ArC) and 190.9 (C=O). Compound 2: the procedure described for the synthesis of 1 was followed using 3-ethoxy-2-hydroxybenzaldehyde (1.66 g, 10 mmol). Work up afforded 2-(benzyloxy)-3-ethoxybenzaldehyde as a white solid which was recrystallized from hexane to afford colourless rod-like crystals (0.86 g, 34%), mp 78–79 °C (lit.^13^ 39–40 °C); *ν*_max_/cm^−1^ 1242 (C–O), 1735 (C=O); *δ*_H_ (600 MHz; CDCl_3_) 1.51 (3H, t, *J* = 7.0 Hz, CH_3_), 4.15 (2H, q, *J* = 7.0 Hz, CH_2_) 5.20 (2H, s, PhCH_2_), 7.11 (1H, t, *J* = 7.9 Hz, ArH), 7.17 (1H, dd, *J* = 8.0, 1.4 Hz, ArH), 7.32–7.43 (6H, m, ArH overlapping) and 10.27 (1H, s, HC=O); *δ*_C_ (150 MHz; CDCl_3_) 15.0 (CH_3_), 64.9 (CH_2_), 76.4 (PhCH_2_), 119.2, 119.4, 124.3, 128.6, 128.7, 128.8, 130.5, 136.7, 151.5, 152.5 (ArC) and 190.4 (C=O). Compound 3: the procedure described for the synthesis of 1 was followed using 5-bromo-2-hydroxybenzaldehyde (2.01 g, 10 mmol). Work up afforded 2-(benzyloxy)-5-bromobenzaldehyde as an off-white solid which was recrystallized from hexane/methanol mixture to afford colourless block crystals (1.66 g, 57%), mp 69–70 °C (lit.^14^ 70–71 °C); *ν*_max_/cm^−1^ 1234 (C–O), 1735 (C=O); *δ*_H_ (400 MHz; CDCl_3_) 5.18 (2H, s, PhCH_2_), 6.95 (1H, d, *J* = 8.9 Hz, 3H), 7.34–7.46 (5H, m, ArH overlapping), 7.60 (1H, dd, *J* = 8.9, 2.8 Hz, 4H), 7.94 (1H, d, *J* = 2.8 Hz, 6H) and 10.46 (1H, s, HC=O); *δ*_C_ (100 MHz; CDCl_3_) 71.0 (PhCH_2_), 114.0, 115.3, 126.6, 127.5, 128.6, 128.9, 131.2, 135.7, 138.3, 160.0 (ArC) and 188.4 (C=O). Compound 4: the procedure described for the synthesis of 1 was followed using 5-chloro-2-hydroxybenzaldehyde (1.57 g, 10 mmol). Work up afforded 2-(benzyloxy)-5-chlorobenzaldehyde as a white solid which was recrystallized from hexane to afford pale brown block crystals (2.13 g, 86%), mp 77–79 °C (lit.^8^ 68–69 °C); *ν*_max_/cm^−1^ 1233 (C–O), 1734 (C=O); *δ*_H_ (400 MHz; CDCl_3_) 5.18 (2H, s, PhCH_2_), 7.00 (1H, d, *J* = 8.8 Hz, 3H), 7.34–7.44 (5H, m, ArH overlapping), 7.46 (1H, dd, *J* = 8.9, 2.8 Hz, 4H), 7.80 (1H, d, *J* = 2.8 Hz, 6H) and 10.48 (1H, s, HC=O); *δ*_C_ (100 MHz; CDCl_3_) 71.0 (PhCH_2_), 114.9, 126.2, 126.8, 127.4, 128.1, 128.6, 128.9, 135.5, 135.7, 159.5 (ArC) and 188.5 (C=O). Compound 5: the procedure described for the synthesis of 1 was followed using 3,5-dibromo-2-hydroxybenzaldehyde (2.80 g, 10 mmol). Work up gave 2-(benzyloxy)-3,5-dibromobenzaldehyde as a white fluffy solid which was recrystallized from hexane/water mixture to afford colourless rod-like crystals (1.62 g, 44%), mp 66–68 °C lit.^15^ 109.5–110.5 °C; *ν*_max_/cm^−1^ 1209 (C–O), 1736 (C=O); *δ*_H_ (400 MHz; CDCl_3_) 5.12 (2H, s, PhCH_2_), 7.38–7.41 (5H, m, ArH overlapping), 7.86 (1H, d, *J* = 2.4 Hz, 6 H), 7.98 (1H, d, *J* = 2.6 Hz, 4H) and 9.96 (1H, s, HC=O); *δ*_C_ (100 MHz; CDCl_3_) 78.13 (PhCH_2_), 118.4, 119.6, 129.0, 129.1, 129.3, 130.5, 132.5, 134.8, 141.5, 157.5 (ArC) and 187.6 (C=O). Compound 6: a mixture of 2-hydroxy-3-methoxybenzaldehyde (3.80 g, 25 mmol), propargyl bromide (1.93 mL, 25.5 mmol) and K_2_CO_3_ (6.9 g, 50 mmol) was heated at 80 °C in acetonitrile (CH_3_CN) for four hours. The solvent was then evaporated and the crude product was dissolved in dichloromethane (CH_2_Cl_2_) and washed with water. The organic layer was dried and the residue recrystallized in hexane/water mixture to obtain 3-methoxy-2-(prop-2-yn-1-yloxy)benzaldehyde as pale brown block crystals (4.37 g, 92%), mp 52–53 °C (lit.^16^ 51–52.5 °C); *ν*_max_/cm^−1^ 1736 (C=O), 3262 (≡CH); *δ*_H_ (400 MHz; CDCl_3_) 2.47 (1H, t, *J* = 2.4 Hz, 3′-CH), 3.90 (3H, s, CH_3_), 4.87 (2H, d, *J* = 2.4 Hz, 1′-CH_2_), 7.16 (2H, q, *J* = 7.8 Hz, 4 and 5H), 7.44 (1H, dd, *J* = 7.0, 2.4 Hz, 6H) and 10.48 (1H, CHO); *δ*_C_ (100 MHz; CDCl_3_) 56.2 (CH_3_O), 61.0 (C-1′), 77.0 (C-3′), 78.4 (C-2′), 117.9, 119.0, 125.0, 131.3, 149.6 and 152.9 (ArC) and 190.7 (C=O).

### 2.3 Methods

#### 2.3.1 Hirshfeld surface analysis

The Hirshfeld surfaces are mapped with *d*_norm_, and 2D fingerprint plots presented in this work were generated using *CrystalExplorer 2.1*.^12^ The 2D plots were shaped by binning (*d*_i_, *d*_e_) pairs in intervals of 0.01 Å and colouring each bin of the resulting 2D histogram as a function of the fraction of surface points in that bin, ranging from blue through green to red. Graphical plots of the molecular Hirshfeld surfaces were mapped with *d*_norm_ using a red-white-blue colour scheme, where red highlights shorter contacts, white is used for contacts around the vdW separation, and blue is for longer contacts.

#### 2.3.2 Quantum chemical calculations

##### Molecular geometry

All calculations done using Gaussian 09D.^17^ All geometry was calculated with the B3LYP^18^ functional, using the 6-311g(d,p)^19^ basis set and an Ultrafine grid. The energy for B3LYP was taken from the same calculation. The M06HF energy was calculated at the same basis set from the B3LYP geometry.

##### Crystal geometry

The structures were optimised using a constrained PBC geometry calculation (with the translation vectors obtained from the CIF files). Energy was calculated for both the B3LYP and M06HF^20^ functionals with the 6-311g(d,p) basis set. The dispersion effects were accounted for using Grimme's type 3 dispersion (DFT–D3)^21^ and the Basis Set Superposition Error (BSSE) was calculated using Gaussian 09's counterpoint method.

#### 2.3.3 Lattice energy calculations

Crystal lattice energies (kJ mol^−1^) were calculated from single-crystal X-ray diffraction data using the atom–atom force field with subdivision of the interaction energies into coulombic, polarization, London dispersion, and Pauli repulsion components (AA-CLP method implemented in the CLP-PIXEL computer program package, ver. 3.0; available from http://www.angelogavezzotti.it).^22^ Default settings were used, and hydrogen atom positions were assigned by the software.

#### 2.3.4 Single-crystal X-ray diffraction

All datasets were collected at 200 K using a Bruker APEX-II CCD diffractometer equipped with graphite monochromated Mo Kα radiation (*λ* = 0.71073 Å). Flack parameters for the non-centrosymmetric structures 1 and 5 are −0.01(15) and 0.015(11), respectively. Data reduction was carried out using the Bruker program SAINT.^23^ A numerical absorption correction SADABS^24^ was applied. The structures of the title compounds were solved using a dual-space algorithm and refined by the full-matrix least-square technique on F2 with anisotropic thermal parameters to describe the thermal motions of all non-hydrogen atoms using the programs SHELXT-2018/2 (ref. 25) and SHELXL-2018/3 (ref. 26) respectively. The hydrogen atoms of the methyl groups were allowed to rotate with a fixed angle around the C–C bonds to best fit the experimental electron density while all other hydrogen atoms were placed at geometrically idealized positions. The methyl hydrogen atoms were assigned isotropic temperature factors equal to 1.5 times the equivalent temperature factor of the parent atom whereas the displacement parameters for the other hydrogen atoms were taken as *U*_iso_(H) = 1.2*U*_eq._(C). Programs: PLATON,^27^ Mercury^28^ and X-Seed.^29^ For structural data and refinement parameters for the title compounds, refer to Table 1.

**Table 1:** Structural data and refinement parameters for compounds 1–6

Compound	1	2	3	4	5	6
CCDC no.	1898081	1895105	1898076	1898075	1898074	1898078
Formula	C_14_H_12_O_2_	C_14_H_16_O_3_	C_14_H_11_BrO_2_	C_14_H_11_ClO_2_	C_14_H_10_Br_2_O_2_	C_11_H_10_O_3_
MW/g mol^−1^	212.24	256.29	291.14	246.68	370.04	190.19
*λ*/Å	0.71073	0.71073	0.71073	0.71073	0.71073	0.71073
*T*/K	200	200	200	200	200	200
Crystal system	Orthorhombic	Monoclinic	Monoclinic	Monoclinic	Orthorhombic	Triclinicic
Space group	*Pna*2_1_	*P*2_1_/*c*	*P*2_1_/*n*	*P*2_1_/*n*	*P*2_1_2_1_2_1_	*P*1̄
*a*/Å	11.5088(5)	15.3768(15)	7.316(4)	4.9219(2)	4.0992(2)	7.7110(4)
*b*/Å	12.9889(6)	4.5578(5)	13.239(10)	16.3089(6)	17.1619(7)	7.9405(4)
*c*/Å	7.2608(4)	19.4729(19)	12.300(8)	14.7245(5)	18.9382(8)	9.1857(5)
*α*/°	90	90	90	90	90	65.896(2)
*β*/°	90	99.697(5)	100.36(3)	99.171(2)	90	85.990(2)
*γ*/°	90	90	90	90	90	70.155(2)
*V*/Å^−3^	1085.39(9)	1345.3(2)	1171.9(13)	1166.84(8)	1332.30(10)	481.36(4)
*D*_calc_/mg m^−3^	1.299	1.265	1.650	1.404	1.845	1.312
*Z*	4	4	4	4	4	2
*μ* (mm^−1^)	0.086	0.087	3.493	0.312	6.074	0.096
*F*(000)	448	544	584	512	720	200
2*θ* (°)	56.6	56.8	56.8	56.6	56.6	56.8
*R* (int)	0.015	0.064	0.027	0.023	0.033	0.015
GOOF	1.05	1.02	1.05	1.05	1.04	1.04
*R*_1_^*a*^ (*I* > 2*σ*(*I*))	0.0295	0.0625	0.0263	0.0337	0.0218	0.0366
w*R*_2_^*b*^ (*I* > 2*σ*(*I*))	0.0822	0.1769	0.0618	0.0880	0.0472	0.1024

### 2.4 Results and discussion

#### 2.4.1 Structural analysis

**Fig. 1:**
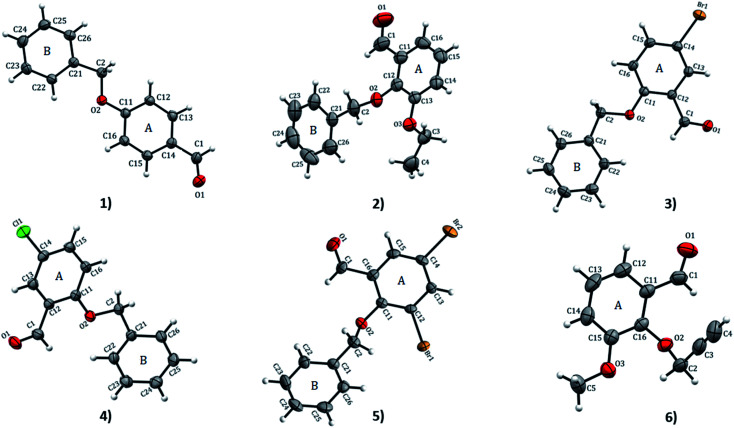
ORTEP view and atom numbering scheme of compound 1–6 with displacement ellipsoids at 50% probability level.

X-ray crystallography X-ray crystallography analyses reveal that 1 and 4 crystallize in orthorhombic with space groups *Pna*2_1_ and *P*2_1_2_1_2_1_, respectively while 2 crystallizes in monoclinic *P*2_1_/*c*; 3 and 5 both crystallize in monoclinic *P*2_1_/*n* space group and 6 crystallizes in triclinic with *P*1̄ space group. ORTEP diagrams of compounds 1–6 drawn with 50% ellipsoid probability are depicted in Fig. 1. The overall molecular conformation in 1–5 can be described by the relative orientation of two phenyl rings (A: C11–C16 atoms, B: C21–C26 atoms) of the benzyloxybenzene core. The structures 2–5 match the position of benzyloxy substituent in the A ring (at the 2-position), while 1 has the benzyloxy substituent at the 4-position. Compound 6 differs from the rest of the structures with the benzyloxy substituent missing. Compound 2 and 6 differ in respect to the ethoxy substituent in the B ring (at 3- and 2-positions, respectively). The molecule of 1 is essentially planar with r.m.s. deviation of 0.0608 Å. In the crystal structures of the compounds, the O⋯O distances range from 2.686(8)–6.380(2) Å. The shortest O⋯O distances is found in 1 while 2 has the longest. The bond-lengths of the A ring in 1–6 lie between 1.374(4) to 1.405(3) Å, the former being at the aromatic carbon flanking the ethoxy substituent in 2, and the latter at the point of substitution of the propargyl (2-propynyl) group in 6 and IPEXEH. The internal bond-lengths in the B ring of the compounds (1–5) range from 1.334(6) to 1.429(5) Å (the bond-lengths reside in C23–C24 and C25–C26 of 2, respectively).

The bond-angles generally agree well with those featuring in analogous structures.^4,11^ These bond-lengths do not vary significantly despite the differing intermolecular interaction patterns observed in various structures.^11^ The geometry about the C=O group is very similar in all the compounds. The bond-angles of substituted benzaldehydes of 1–6 agree well with the mean values of relevant bond-angles obtained with MOGUL (version 1.75, Nov 2018)^30^ from searches based on related molecular fragments on the Cambridge Structural Database (CSD version 5.40, Nov 2018).^10^ The dihedral angle between the A ring and linear propargyloxy group in 6 is the largest angle equal to 68.37° which is vastly different from 61.77° of IPEXEH. The dihedral angle between the A and B rings vary significantly with 1 having the smallest angle equal to 5.40° (while for DUTRIU, DUTRIU01 and DUTRIU02 the A/B dihedral angle is 5.23°,5.86° and 4.97° respectively). In all the compounds with the exception of compound 5; the carbonyl, methoxy and ethoxy groups as well as the halogens lie in the same plane of the A ring. In 5, the A ring is slightly distorted and consequently the two substituted Br1 and Br2 atoms both lie just outside the plane of the ring.

**Fig. 2:**
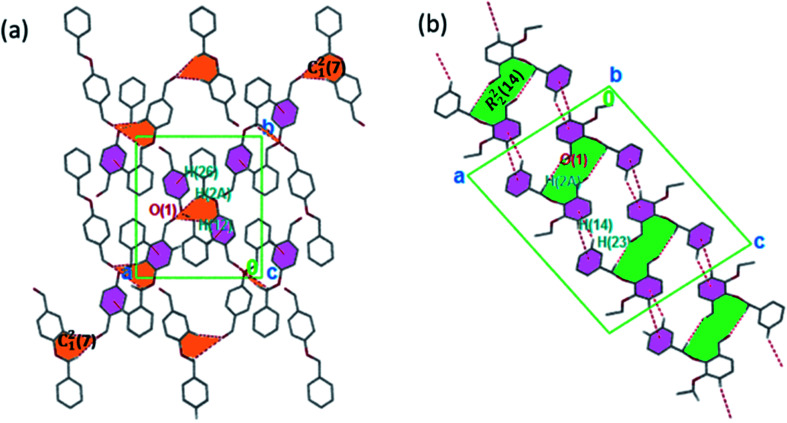
(a) 3D parallel chains in 1 running along the [001] direction; (b) a stair-case like supramolecular framework in 2 propagating along the [010] direction. The crystalline solids are generated *via* C–H⋯O and C–H⋯π interactions. Hydrogen atoms not involved in hydrogen bonding have been omitted.

**Fig. 3:**
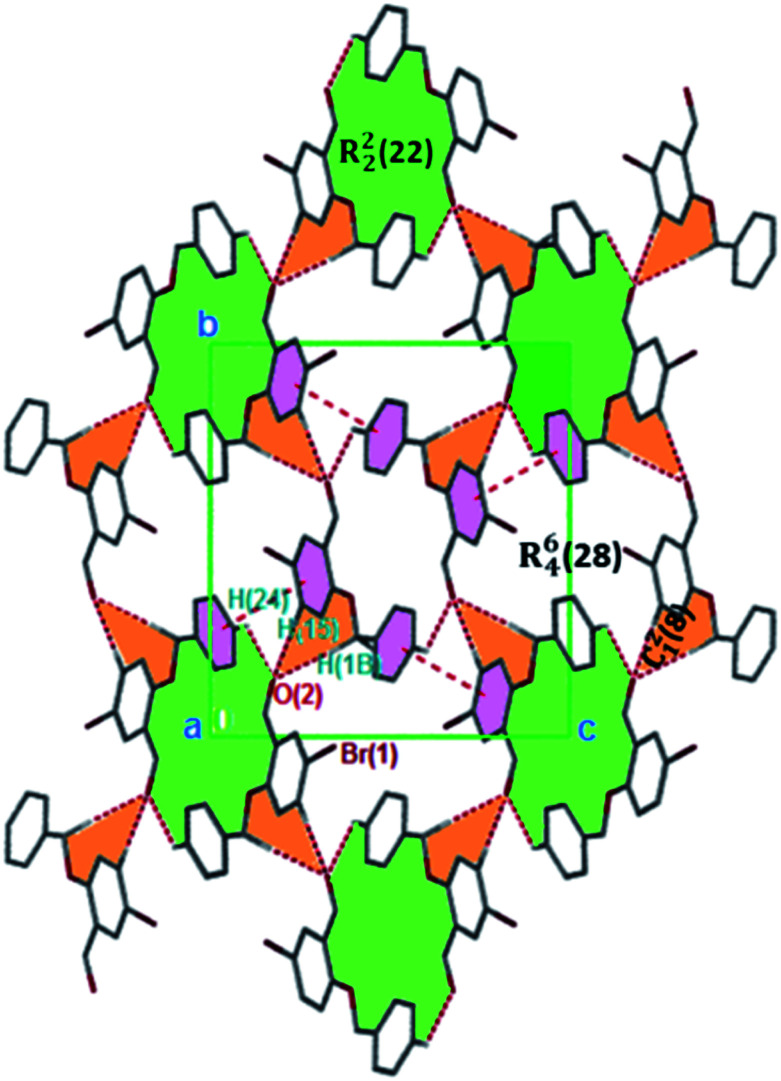
Monomeric 2D sheets in 3 running along the [100] plane. The crystalline solids are generated *via* C–H⋯O and π⋯π interactions. Hydrogen atoms not involved in hydrogen bonding have been omitted.

**Fig. 4:**
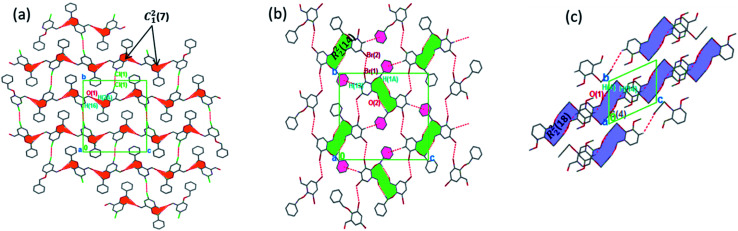
(a) 3D parallel chains in 4 running along the [100] direction; (b) 2D supramolecular framework in 5 propagating along the [100] plane; (c) parallel networks in 6 propagating along the [100] direction. Hydrogen atoms not involved in hydrogen bonding have been omitted.

The torsion angle C21–C2–O2–C11 of −176.4(1)°, +170.5(2)°, −177.1(2)°, −176.0(2)°, and +177.2(1)° in 1–5 displays an anti-configuration of molecules about the C2–O2 bond while the torsion angle C3–C2–O2–C12 in 6 is −66.77°. The crystal packing in 1–6 (Fig. 2–4) is secured by intermolecular contacts. The supramolecular assembly in these structures can be readily seen as substructures of lower dimensionality with synthons as the building blocks. In 1, the phenyl ring carbon atom C2 in the molecule at (*x*, *y*, *z*) acts as a hydrogen bond donor to the carbonyl oxygen atom O1 at (1 − *x*, −1/2 + *y*, 1/2 − *z*). The propagation of dimers *via* intermolecular C2–H2A⋯O1, C2–H2B⋯O1 hydrogen bonds and C26–H26⋯π(arene) (2.790 Å) in 1 generates C_1_^2^(7) parallel chains to which their combination form three-dimensional polymeric chains (comparably to DUTRIU, DUTRIU01 and DUTRIU02) running along the [001] direction (Fig. 2a). In 2, the benzyloxy carbon atom C2 in the molecule acts as a hydrogen bond donor to the carbonyl oxygen atom O1 in the molecule at (1 − *x*, −1/2 + *y*, 1/2 − *z*). The structure is generated *via* hydrogen bonding interactions to give rise to the formation of a dimeric R_2_^2^(14) synthon propagating along the [100] direction, forming 2D parallel columns (Fig. 2b) propagating along the [010] plane. Additionally the packing arrangement of the compound is further generated through intermolecular C14–H14⋯π(arene) hydrogen bonding (2.768 Å) and π(lone pair)–π(lone pair) stacking interactions (3.214 Å). Despite the similarity between 3 and 5 in terms of their molecular geometries, there are subtle differences in their supramolecular self-organization. In the crystal structures, the compounds 3 and 5 adopt a dimeric form of C15–H15⋯O2 (O2 at 3/2 − *x*, −1/2 + *y*, 1/2 − *z*) and C1–H1A⋯O2 (O2 at −1/2 + *x*, 3/2 − *y*, 1 − *z*) hydrogen bonding with distance of 2.463 and 2.475 Å (Table 2), respectively.

**Table 2:** Hydrogen bonding geometry of compounds 1–6^a^

	D–H⋯A	*d*(D–H)	*d*(H⋯A)	*d*(D⋯A)	*L*(D–H⋯A)	*L* ^s^ b
1	C2–H2B⋯O1^(i)^	0.99	2.55	3.343(2)	142	5.40
	C26–H26⋯Cg(1)^(ii)^	0.95	2.79	3.657(5)	152	
2	C2–H2A⋯O1^(iii)^	0.99	2.53	3.414(1)	149	22.83
	C14–H14⋯Cg(2)^(iv)^	0.95	2.77	3.657(1)	156	
3	C15–H15⋯O1^(v)^	0.95	2.46	3.408(4)	173	21.66
4	C2–H2A⋯O1^(vi)^	0.99	2.67	3.620(2)	162	23.48
	C16–H16⋯O1^(vii)^	0.95	2.64	3.351(2)	132	
5	C2–H2A⋯O1^(viii)^	0.99	2.48	3.447(8)	167	20.69
	C13–H13⋯Cg(2)^(ix)^	0.95	2.84	3.773(8)	169	
6	C4–H4⋯O1^(x)^	0.95	2.32	3.198(6)	154	68.37
	C4–H14⋯π^(xi)^	0.95	2.81	3.653(6)	148	

a Cg(1) and Cg(2) are the centroids of A and B rings, respectively.

b A/B ring dihedral angles (in degree). Symmetry codes: (i) −1/2 + *x*, 3/2 − *y*, *z*; (ii) 1/2 + *x*, 1/2 − *y*, *z*; (iii) 1 − *x*, −1/2 − *y*, 1/2 − *z*; (iv) *x*, 1/2 − *y*, 1/2 + *z*; (v) 3/2 − *x*, −1/2 + *y*, −*z*; (vi) −1/2 + *x*, 1/2 − *y*, −1/2 + *z*; (vii) −1/2 + *x*, 1/2 − *y*, 1/2 + *z*; (viii) −1/2 + *x*, 3/2 − *y*, 1 − *z*; (ix) 1/2 − *x*, 1 − *y*, −1/2 + *z*; (x) −*x*, 1 − *y*, 2 − *z*; (xi) −*x*, 1 − *y*, 1 − *z*.

The formation of dimers *via* intermolecular contacts of the nature C1–H1B⋯O2 and C1–H1A⋯O2 are respectively seen in 3 and 5. These hydrogen bonds generate C_1_^2^(8), R_2_^2^(22) and R_4_^6^(28) graph-set motifs respectively propagate along the [100], [011] and [010] directions in 3 to build three-dimensional supramolecular networks and R_2_^2^(14) motif running along the [001] plane in 5 forming honeycomb sheets running along the [100] direction (Fig. 3 and 4b). The structure 3 is further stabilized through π–π (3.352 Å) stacking interactions (the benzyl ring (A) carbon atom C16 in the molecule at (*x*, *y*, *z*) acts as a donor to the centroid of another benzyl ring (B)) while 5 is stabilized through C13–H13⋯π(arene) (2.837 Å) hydrogen bond and (lone pair)–π(arene) (3.090 Å) stacking interactions. In compound 4, perpendicular chains (Fig. 4a) running along the [100] plane are linked *via* C2–H2A⋯O1 (O1 at −1/2 + *x*, 1/2 − *y*, −1/2 + *z*) and C16–H16⋯O1 (O1 at −1/2 + *x*, 1/2 − *y*, 1/2 + *z*) hydrogen bond (Table 2) offering a C_1_^2^(7) parallel chains similar to compound 1. In the presence C–H⋯O hydrogen bond, unlike compound 3, the crystal packings in 4 and 5 are further generated by Cl1⋯Cl1 with interatomic distance 3.227 Å (Cl1 at −1 − *x*, −*y*, 1 − *z*) and Br1⋯Br2 with interatomic distance 3.515 Å (−*x*, −1/2 + *y*, 3/2 − *z*) type I halogen bonding interactions, respectively. In 6, the carbonyl oxygen O1 atom in the molecule at (*x*, *y*, *z*) acts as hydrogen bond acceptor from the alkyne carbon C4 at (−*x*, 1 − *y*, 2 − *z*), thus constructing chair conformations propagating along the [100] direction (Fig. 4c). The structure 6 is generated through C4–H4⋯O1 and C4–H14⋯π(alkyne) hydrogen bonding interactions (Table 2) constructing a chair-like R_2_^2^(18) synthon, which is similar to IPEXEH, to give rise to the formation of supramolecular networks.

#### 2.4.2 Hirshfeld surface and lattice energy analyses

**Fig. 5:**
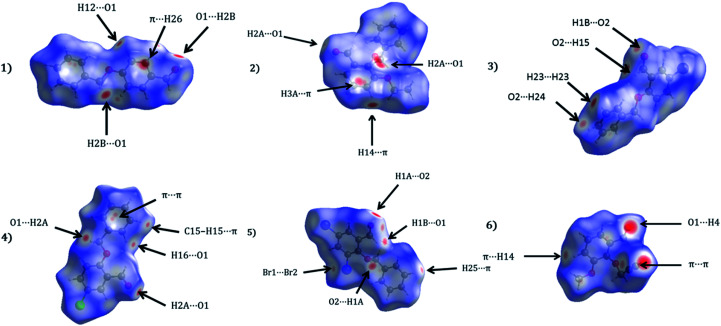
Hirshfeld surfaces mapped with *d*_norm_ for the title compounds (1–6).

The Hirshfeld surfaces of 1–6 are shown in Fig. 5, illustrating surfaces that have been mapped over *d*_norm_. The surfaces are presented as transparent to permit visualization of the substituted benzaldehyde moiety around which they were calculated. The colour codes from red for short *d*_norm_ ranges to blue for long ranges were presented. The dominant interactions in the title derivatives can be seen as the bright red areas. Fig. 5 effectively summarizes the Hirshfeld surfaces as large/bright red areas are indicative of C–H⋯O and, C–H⋯π hydrogen bonding interactions.

**Fig. 6:**
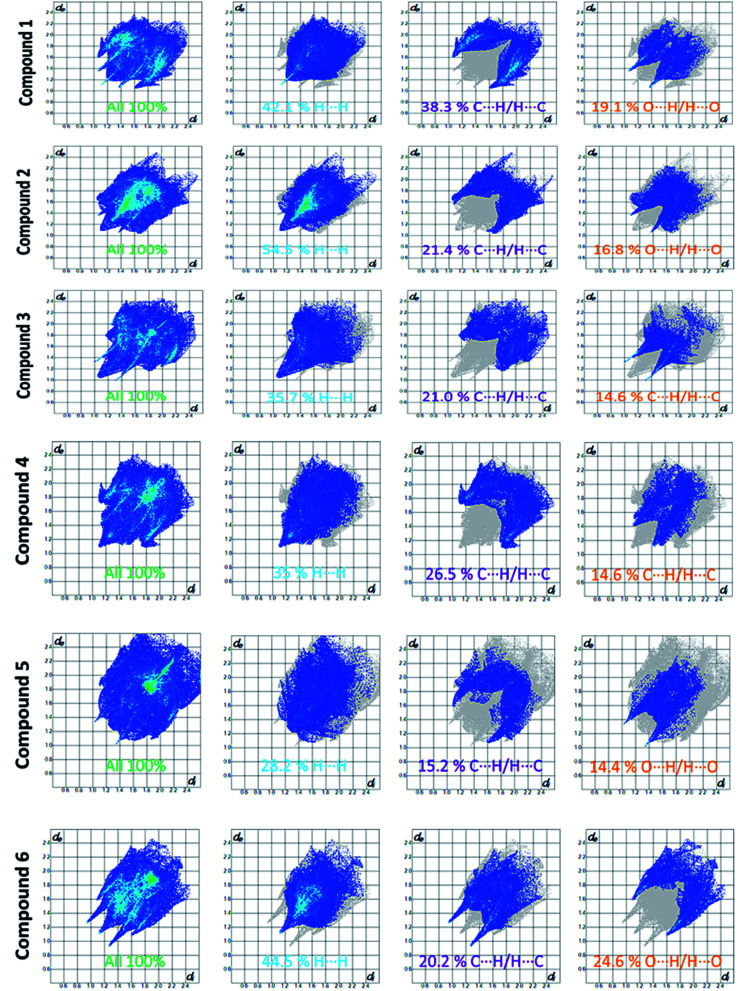
Fingerprint plots: full, H⋯H, C⋯H/H⋯C and O⋯H/H⋯O contacts for 1–6 displaying percentages of contacts contributed to the total Hirshfeld surface area of the compounds.

**Fig. 7:**
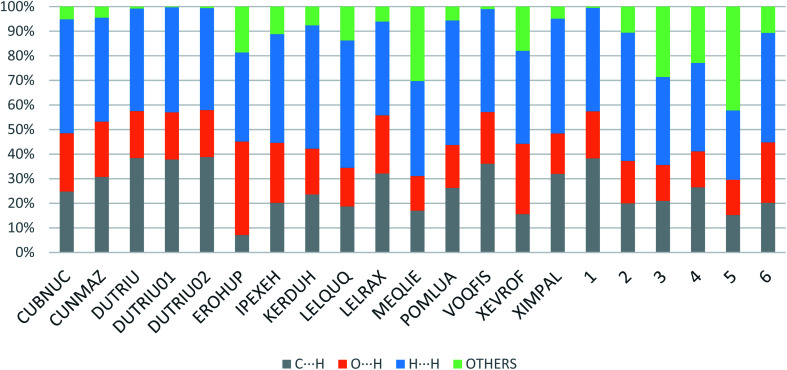
Relative contributions of various intermolecular contacts to the Hirshfeld surface area in 1–6 and some related structures retrieved from the CSD.

**Fig. 8:**
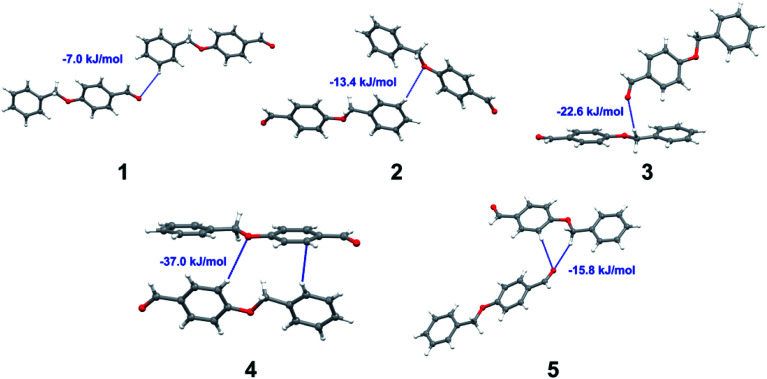
Molecular pairs (1–5) along with their interaction energies (values in blue) in 1.

**Fig. 9:**
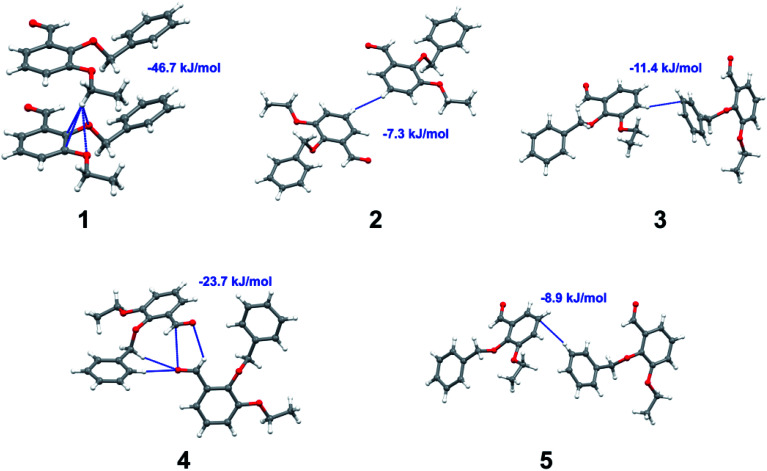
Molecular pairs (1–5) along with their interaction energies (values in blue) in 2.

**Fig. 10:**
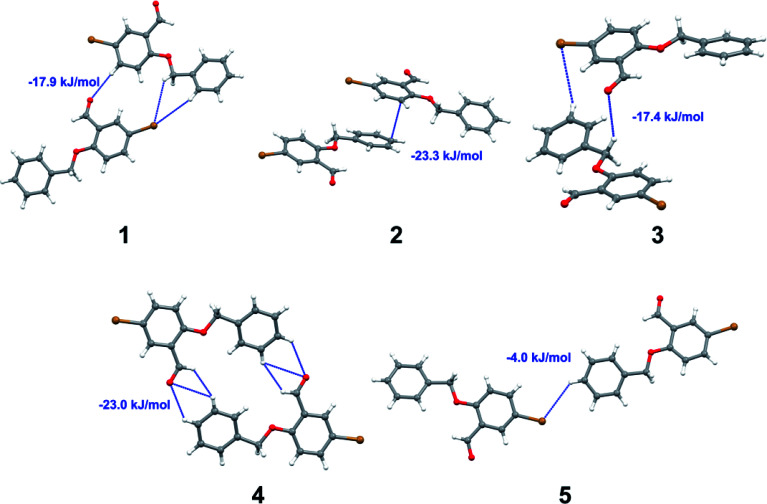
Molecular pairs (1–5) along with their interaction energies (values in blue) in 3.

**Fig. 11:**
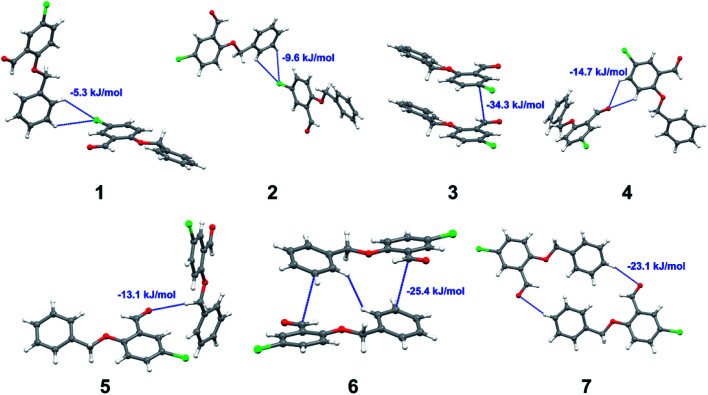
Molecular pairs (1–7) along with their interaction energies (values in blue) in 4.

**Fig. 12:**
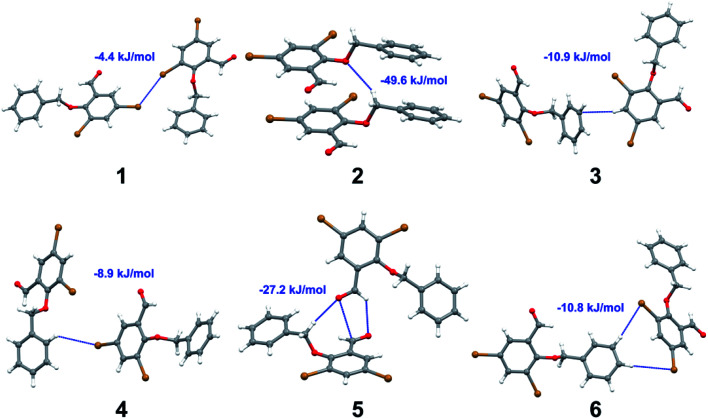
Molecular pairs (1–6) along with their interaction energies (values in blue) in 5.

**Fig. 13:**
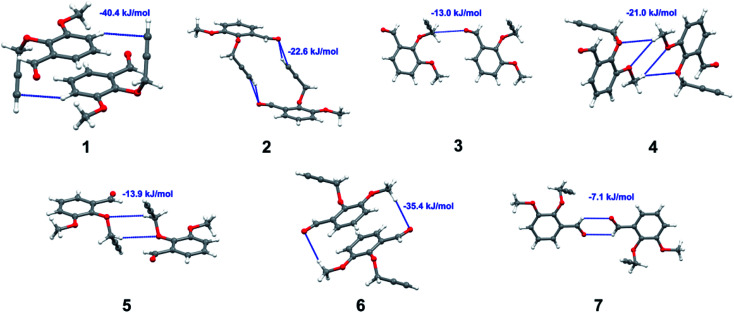
Molecular pairs (1–7) along with their interaction energies (values in blue) in 6.

Apart from hydrogen bonding, the π–π stacking interactions are seen on the *d*_norm_ surfaces as small red areas in 4 and large red areas in 6. The other trivial ranges of noticeable red spots and light-white regions are demonstrative of weaker and longer contacts other than hydrogen bonds. In addition, Fig. 6 depict the two-dimensional fingerprint plots that decompose to highlight particular atoms pair close contacts and in Fig. 7 the relative contributions of individual intermolecular interactions to the Hirshfeld surfaces area are shown. In 1, the O⋯H/H⋯O intermolecular interactions appear as a pair of symmetrical large sharp spikes in the fingerprint plots with *d*_i_ + *d*_e_ = 2.45 Å which comprise 19.1% of the total Hirshfeld surfaces area. The C⋯H/H⋯C interactions comprise 38.3% of the total Hirshfeld surfaces and represent two small wings in the region of (1.09 Å, 1.57 Å). Furthermore, the H⋯H interactions are presented in the distribution of scattered points in the fingerprint plots, which split-up to *d*_i_ = *d*_e_ = 1.10 Å and comprise 42.1% of the total Hirshfeld surfaces. Contrast to 1, the O⋯H/H⋯O intermolecular interactions are longer in 2 (*d*_i_ + *d*_e_ = 2.52 Å), 4 (*d*_i_ + *d*_e_ = 2.65 Å) and 5 (*d*_i_ + *d*_e_ = 2.38 Å) but shorter in 3 (*d*_i_ + *d*_e_ = 2.31 Å) and 6 (*d*_i_ + *d*_e_ = 2.12 Å), respectively, with the following percentages 16.8%, 14.6%, 14.4%, 14.6% and 24.6% to the total Hirshfeld surfaces, respectively. However, the C⋯H/H⋯C interactions are displayed in two sharp spikes in the fingerprint plots, which spread up to the longer distance of *d*_i_ + *d*_e_ = 2.73 Å, 2.78 Å, 2.65 Å, 2.71 Å and 2.73 Å for 2, 3, 4, 5 and 6, respectively, and contribute 21.4%, 21.0%, 26.5%, 15.2% and 20.2% to the total Hirshfeld surfaces, respectively. The H⋯H interactions have more significant contribution to the total Hirshfeld surfaces in 1 (42.1%) and 6 (44.5%) when compared with compounds 2–5, which spread up to *d*_i_ = *d*_e_ = 2.25 Å and *d*_i_ = *d*_e_ = 2.2 Å, respectively. The most significant differences amongst 1–6 is the presence of π(lone pair)⋯π(arene) stacking interactions in 2, 3 and 5. The π–π stacking interactions are longer in 2 (*d*_i_ + *d*_e_ = 2.85 Å) and in 3 (*d*_i_ + *d*_e_ = 2.3 Å) but closer in 5 (*d*_i_ + *d*_e_ = 3.6 Å), and the proportions of the total Hirshfeld surfaces are 4.2%, 8.2% and 8.5%, respectively. The relative contribution of different interactions to the Hirshfeld surfaces was calculated for 1–6 as well as a few benzyloxy-benzaldehyde derivatives retrieved from the CSD. It is evident in Fig. 7 that the molecular interactions in the title compounds and benzyloxybenzaldehyde derivatives mentioned earlier are pre-dominantly of H⋯H, C⋯H and O⋯H types, which can account for 87% of the Hirshfeld surface area. Table 3 lists the lattice energies obtained from AA-CLP^20^ and *ab initio* DFT calculations. Quantitative comparison between the AA-CLP, B3LYP and M06HF methods was made possible to explore how similar, or different, are the models. However, the comparison is limited to energies for small numbers of molecular pairs. A simple regression analysis reveals that overall *E*_Tot_ (AA-CLP) = 2.380 *E*_Tot_ (B3LYP) and *E*_Tot_ (AA-CLP) = 2.232 *E*_Tot_ (M06HF), although differences between the two can be as large as +1.1 and +1.2 kJ mol^−1^ for B3LYP and M06HF (POMLUA) respectively. This means that the lattice energy calculated by AA-CLP (−44.8 kJ mol^−1^) coincide with both B3LYP (−45.7 kJ mol^−1^) and M06HF (−45.8 kJ mol^−1^) methods only for POMLUA. The DFT total energy (*E*_Tot_) calculations showed a direct relationship with thermal strengths of the title compounds (Table S1 and Fig. S1†). The molecular pair interaction energies for the title compounds are shown in Table S3.† Molecular pairs of 1 (1–5) extracted from crystal structure along with their respective interaction energies are shown in Fig. 8. The maximum stabilization to the crystal structure comes from C–H⋯O intermolecular interaction involving H23 with O1. The stabilization energy of the pair is −7.0 kJ mol^−1^ (motif 1) obtained using *crystalexplorer* v17.5. Another molecular pair (motif 2) has interaction energy of −13.4 kJ mol^−1^ also involves C–H⋯O intermolecular interaction involving H25 with O2. Motif 3 involves H2B and O1 with stabilization energy being −26.6 kJ mol^−1^. The next stabilized pair (motif 4) show C–H⋯π intermolecular interaction between H26 and C15 atoms with stabilization energy of −37.0 kJ mol^−1^. Last molecular pair 5 involves the interaction of H12 with O1. This pair also involves the interaction of H2A with O1 having interaction energy contributing towards the stability of crystal packing. The interaction for motif (1–5) in 1 is primarily dispersive in nature (Table S3†). The most stabilized molecular pairs (1–5) of 2 along with their stabilization energies are shown in Fig. 9. The most stabilized molecular pair (motif 1) in 2 shows the presence of C–H⋯π involving H3A with C12, C13 (of the A ring), O2 and O3 and provides stabilization of −46.7 kJ mol^−1^. The next stabilized pair (motif 2) shows H⋯H (involving H15 interacting with H15 atom) resulting in a stabilization energy of −7.3 kJ mol^−1^. The stabilized pair (motif 3) involves C–H⋯π hydrogen bonding (involving H14 with C23) having an interaction energy of −11.4 kJ mol^−1^. The second most stabilized pair is motif 4 which shows the presence of C–H⋯O (involving O1 interacting with H1, H2A and H22) and lone pair⋯lone pair (between C1 and O1) forming dimer having an interaction energy of −23.7 kJ mol^−1^. Molecular pair 5 shows the presence of C–H⋯π (involving H23 with C15) having an interaction energy of −8.9 kJ mol^−1^ that contributes towards the stability of crystal packing. The interaction for motif (1–5) in 2 is similar to 1 and it is primarily dispersive in nature. The extracted molecular pairs (1–5) of 3 are shown in Fig. 10 along with their stabilization energies. The stabilized molecular pair (motif 1) shows the presence of C–H⋯O hydrogen bonding (involving O1 acceptor with H15) and also the presence of bifurcated acceptor atom involved in C–H⋯Br halogen bonding (involving Br1 interacting with both H2B and H26) with interaction energy of −17.9 kJ mol^−1^. The most stabilized pair (motif 2) shows the presence of π–π (involving C16 with C24 atoms of the A and B rings, respectively) to form a dimer having an interaction energy of −23.3 kJ mol^−1^. Molecular pair 3 shows the presence of both C–H⋯O/Br hydrogen/halogen bonding (involving H2B with O1 and H24 interacting with Br1) resulting in a stabilization energy of −17.4 kJ mol^−1^. The second most stabilized pair (motif 4) shows the presence of bifurcated acceptor atom involved in C–H⋯O hydrogen bonding twice (involving O1 interacting with both H23 and H24) and H⋯H (involving H2 with H25) to generate a molecular pair having an interaction energy of −23.0 kJ mol^−1^. Finally, the least stabilized pair shows C–H⋯Br interaction (involving H24 with Br1) having an interaction energy of −4.0 kJ mol^−1^ which provides additional stabilization to the crystal packing. The major contribution to the stabilization in 3 comes from dispersion component. Molecular pairs of 4 (1–7) extracted from crystal structure along with their respective interaction energies are shown in Fig. 11. The interaction for motif (1–5) in 4 is primarily repulsive in nature. The minimum stabilization to the crystal involves molecular stacking to generate dimers and a bifurcated acceptor atom involved in C–H⋯Cl halogen bonding (involving Cl1 interacting with H24 and H25) in both motifs 1 & 2. The stabilization energies of these pairs are −5.3 and −9.6 kJ mol^−1^, respectively and the combined nature of these interactions is mainly dispersive in nature. The most stabilized pair (motif 3) shows the presence of lone pair–π interaction (involving C1 with C14 of the A ring) having an interaction energy of −34.3 kJ mol^−1^ and is dispersive in nature. Motif 4 shows the presence of a bifurcated interaction (involving O1 with both H15 and H16) to generate a molecular pair having an interaction energy of −14.7 kJ mol^−1^. Another molecular pair (motif 5) in 4 shows the presence of C–H⋯O hydrogen bonding (involving H2A with O1) forming a dimer with interaction energy of −13.1 kJ mol^−1^. Molecular pair 6 having an interaction energy of −25.4 kJ mol^−1^ shows lone pair–π (involved in C1 and C23 of the B ring) and H⋯H interaction (involving H22 with H22) to contribute towards the stability of crystal packing. Molecular pair 7 involves two H24 interactions with O1 to give stabilization energy of −23.1 kJ mol^−1^. The molecular pairs (1–6) which provide maximum stabilization to the packing in 5 are shown in Fig. 12. For both structures 5 and 6 the interaction for motifs (1–6) and motif (1–7) are predominantly repulsive in nature. The least stabilized molecular pair in 5 involves type I Br⋯Br intermolecular interaction, (involving Br1 with Br2) having an interaction energy of −4.4 kJ mol^−1^ with major contribution from repulsion component. The next most stabilized pair involves C–H⋯O hydrogen bonding (involving H2B with O2) resulting in a stabilization energy of −49.6 kJ mol^−1^. Molecular pair 3 shows the presence of C–H⋯π (involving H13 and C13 of the B ring) having an interaction energy of −10.9 kJ mol^−1^. The next two stabilized pairs shows C–H⋯Br hydrogen bonding, motif 4 involves H26 interacting with Br2 resulting in interaction energy of −8.9 kJ mol^−1^ whereas motif 5 shows the presence of bifurcated interaction (involving O1 interacting with both C2 and H2A) forming dimer having an interaction energy of −27.2 kJ mol^−1^. Last stabilized pair involves C–H⋯Br halogen bonding (involving H23 with Br1 and H24 with Br2) interaction forming a dimer having interaction energy of −10.8 kJ mol^−1^. Molecular pairs (1–7) providing significant contribution towards the stabilization along with their interaction energies for 6 are shown in Fig. 13. The molecular pair with maximum energy stabilization (motif 1) shows the presence of two C–H⋯π hydrogen bonds (H14 interacting with C4) resulting in interaction energy of −40.4 kJ mol^−1^. The next stabilized pair (motif 2) shows the presence of lone pair–π interaction (involving C1 with O1 of the carbonyl) and also C–H⋯O (involving H4 with O1) having interaction energy of −22.6 kJ mol^−1^ and is dispersive in nature. Motif 3 shows the presence of a lone pair–π interaction between O1 (carbonyl oxygen) and C2 with interaction energy of −13.0 kJ mol^−1^. Motif 4 shows the presence of bifurcated donor atom (involving H5C with both O2 and O3) to generate a molecular pair having an interaction energy of −21.0 kJ mol^−1^. Another molecular pair (motif 5) in 6 shows the presence of C–H⋯O hydrogen bonding (involving H2A with O2) forming a dimer with interaction energy of −13.9 kJ mol^−1^. Molecular pair 6 having an interaction energy of −25.4 kJ mol^−1^ shows C–H⋯O (involved in O1 with H5C) to contribute towards the stability of crystal packing. Molecular pair 7 involves two H1 interactions with O1 to give the least stabilization energy of −7.1 kJ mol^−1^.

**Table 3:** Crystal lattice energies (kJ mol^−1^) calculated using AA-CLP and *ab initio* DFT methods for various compounds

Compound	*E*_Ele_	*E*_Pol_	*E*_Disp_	*E*_Ex-rep_	*E*_Tot_	*E*_Tot_ a	*E*_Tot_ b
1	−28.1	−19.2	−126.4	50.6	−126.3	−48.9	−56.4
2	−20.2	−25.2	−136.1	50.7	−130.8	−39.7	−48.1
3	−16.0	−16.8	−148.6	59.0	−122.4	−32.2	−40.4
4	−23.5	−18.4	−137.2	51.1	−127.9	−36.6	−44.8
5	−20.6	−14.1	−156.2	65.0	−125.8	−25.9	−35.0
6	−23.5	−20.1	−101.9	51.3	−94.20	−45.4	−56.3
COBNUC	−33.5	−22.3	−118.7	37.4	−137.0	−67.7	−75.8
CUNMAZ	−36.2	−26.5	−128.9	61.1	−130.5	−28.2	−25.4
DUTRIU	−26.3	−20.3	−123.8	41.9	−131.8	−54.3	−61.8
DUTRIU01	−24.2	−19.3	−111.2	31.5	−126.3	−48.9	−56.3
DUTRIU02	−27.4	−20.6	−128.0	45.6	−133.6	−56.2	−48.9
EROHUP	−35.3	−33.4	−157.9	61.0	−165.7	−68.1	−17.8
KERDUH	−23.5	−24.7	−133.6	46.7	−135.1	−52.1	−57.0
IPEXEH	−22.7	−21.1	−102.7	43.6	−102.9	−56.0	−63.3
LELQUQ	−29.1	−27.8	−123.0	43.4	−136.6	−49.1	−67.5
LELRAX	−19.2	−29.3	−131.3	42.7	−137.0	−53.2	−62.0
MEQLIE	−18.4	−18.4	−122.9	32.9	−126.8	−59.7	−22.5
POMLUA	−4.20	−9.60	−35.10	11.5	−44.60	−45.7	−45.8
VOQFIS	−21.0	−18.5	−113.4	39.9	−113.0	−35.1	−37.6
XEVROF	−34.4	−37.1	−132.2	50.9	−153.2	−57.2	−50.7
XIMPAL	−25.2	−19.1	−117.9	36.3	−125.9	−52.9	−59.8

a DFT (B3LYP) and;

b DFT (M06HF).

## 3 Conclusions

In summary, in our effort to explore the role played by these intermolecular contacts in the self-assembly of crystal structures we have studied six related benzaldehyde derivatives by single crystal X-ray diffraction. It is observed that the carbonyl group generates hydrogen bonded motifs in all compounds studied here and their analogous structures (DUTRIU, DUTRIU01, DUTRIU02 and IPEXEH, shown in Fig. S8†). These crystalline solid materials demonstrate how self-regulating the various weak interactions such as C–H⋯O hydrogen bonding, π–π and lone pair–π stacking, and type I halogen–halogen interactions which complement each other in crystal packing. Furthermore, hydrogen bonding and π–π intermolecular interactions engineered or manoeuvred themselves abruptly but in a cooperative fashion to influence the out of plane molecular stacking. The differences in crystal packing are represented by variation of substitution positions in the compounds. Interestingly, compounds 3 and 4 are isomorphous but their crystal packing is vastly different. Considering self-organization systems of this manner, the study in the field of photo-induced dimerization and crystal engineering in general looks promising. The crystal packing of all the compounds has been analysed using both Hirshfeld surface and theoretical methods. The total energy showed a direct relationship with thermal strengths (“melts”) of the title compounds. The structures studied in this work assisted us to appreciate the influence of intermolecular contacts in constructing supramolecular systems in the solid state.

## Conflicts of interest

There are no conflicts to declare.

## Supplementary Material

RA-010-C9RA10752E-s001

RA-010-C9RA10752E-s002
