# Supplementary material for: Exploring intermolecular contacts in multi-substituted benzaldehyde derivatives: X-ray, Hirshfeld surface and lattice energy analyses
Source: RSC Adv. 2020 Apr 29;10(29):16861–74. doi: 10.1039/c9ra10752e (PMC9053169; doi:10.1039/c9ra10752e)
Supplement: RA-010-C9RA10752E-s001 [file RA-010-C9RA10752E-s001.pdf]

Electronic Supplementary Material (ESI) for RSC Advances.
This journal is © The Royal Society of Chemistry 2020

Electronic Supplementary Information (ESI) for CrystEngComm.
This journal is © The Royal Society of Chemistry 2019

Electronic Supplementary Information

Exploring intermolecular contacts in multi-substituted benzaldehyde derivatives: X-ray, Hirshfeld surface and Lattice energy analyses

Siya T. Hulushe,*[a] Meloddy H. Manyeruke,[a] Marcel Louzada,[a] Sergei Rigin,[b] Eric C. Hosten,[c] and Gareth M. Watkins,[a]

[a]Department of Chemistry, Rhodes University, P.O. Box 94, Grahamstown, South Africa.

[b]Department of Chemistry, New Mexico Highlands University, Las Vegas, New Mexico, 87701, USA

[c]Department of Chemistry, Nelson Mandela University, PO Box 77000, Port Elizabeth 6031, South Africa.

Corresponding Author's email: g11h7156@campus.ru.ac.za

Table S1. Table showing relationship between AA-CLP, DFT total energy (E_{tot}) calculations and thermal strengths of the title compounds.

Compound	E_{Ele}	E_{Pol}	E_{Disp}	E_{Ex-rep}	E_{Tot}	E_{Tot} [a]	E_{Tot} [b]	Temp. / °C
**1**	-28.1	-19.2	-126.4	50.6	-126.3	-48.9	-56.4	52
**2**	-20.2	-25.2	-136.1	50.7	-130.8	-39.7	-48.1	69
**3**	-16.0	-16.8	-148.6	59.0	-122.4	-32.2	-40.4	79
**4**	-23.5	-18.4	-137.2	51.1	-127.9	-36.6	-44.8	70
**5**	-20.6	-14.1	-156.2	65.0	-125.8	-25.9	-35.0	100
**6**	-23.5	-20.1	-101.9	51.3	-94.20	-45.4	-56.3	67

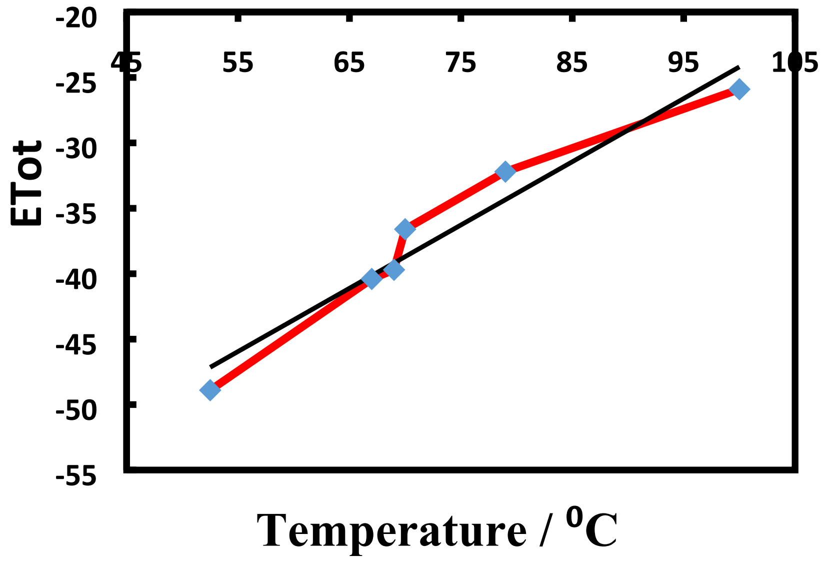

Fig. S1. Showing a direct relationship between DFT total energy (E_{tot}) calculations and thermal strengths of the title compounds

Table S2a. Crystal lattice energies (kJ/mol) calculated using B3LYP DFT methods for various compounds.

			B3LYP		
**Compound**	**LE (a.u)**	**LE (eV)**	**LE (eV/molecule)**	**LE (kJ/molecule)**	**LE (kJ/mole)**
**1**	-0.130407103	-3.548559839	-0.50693712	-8.12215E-23	-4.89E+01
**2**	-0.151038133	-4.109959052	-0.410995905	-6.58498E-23	-3.97E+01
**3**	-0.110461332	-3.005807496	-0.333978611	-5.35101E-23	-3.22E+01
**4**	-0.111610508	-3.037078164	-0.37963477	-6.08251E-23	-3.66E+01
**5**	-0.088843241	-2.417548971	-0.268616552	-4.30377E-23	-2.59E+01
**6**	-0.086513102	-2.354142624	-0.470828525	-7.54361E-23	-4.54E+01
**CUBNUC**	-0.2062925	-5.613507734	-0.701688467	-1.12425E-22	-6.77E+01
**CUNMAZ**	-0.03223413	-0.877135805	-0.292378602	-4.68449E-23	-2.82E+01
**DUTRIU**	-0.144714227	-3.937876711	-0.562553816	-9.01324E-23	-5.43E+01
**DUTRIU01**	-0.130339764	-3.546727454	-0.506675351	-8.11795E-23	-4.89E+01
**DUTRIU02**	-0.149801078	-4.076297046	-0.582328149	-9.33006E-23	-5.62E+01
**EROHUP**	-0.233339889	-6.349505053	-0.705500561	-1.13035E-22	-6.81E+01
**KERDUH**	-0.138977571	-3.781774287	-0.54025347	-8.65594E-23	-5.21E+01
**IPEXEH**	-0.255785667	-6.96028609	-0.580023841	-9.29314E-23	-5.60E+01
**LELQUQ**	-0.149723125	-4.074175844	-0.50927198	-8.15956E-23	-4.91E+01
**LELRAX**	-0.121507167	-3.306380115	-0.551063353	-8.82914E-23	-5.32E+01
**MEQLIE**	-0.068263333	-1.857540869	-0.61918029	-9.92051E-23	-5.97E+01
**POMLUA**	-0.121892	-3.316851969	-0.473835996	-7.5918E-23	-4.57E+01
**VOQFIS**	-0.0801145	-2.180027705	-0.363337951	-5.8214E-23	-3.51E+01
**XEVROF**	-0.00036302	-0.00987829	-0.059269739	-9.4962E-23	-5.72E+01
**XIMPAL**	-0.16130975	-4.389464131	-0.548683016	-8.791E-23	-5.29E+01

Table S2b. Crystal lattice energies (kJ/mol) calculated using M06-HF DFT methods for various compounds.

	M06-HF				
	**LE (a.u)**	**LE (eV)**	**LE (eV/molecule)**	**LE (kJ/molecule)**	**LE (kJ/mole)**
**1**	-0.150440454	-4.093695378	-0.584813625	-9.37E-23	-5.64E+01
**2**	-0.183134529	-4.983346922	-0.498334692	-7.98E-23	-4.81E+01
**3**	-0.138380373	-3.765523691	-0.418391521	-6.70E-23	-4.04E+01
**4**	-0.136492018	-3.714138885	-0.464267361	-7.44E-23	-4.48E+01
**5**	-0.11998112	-3.264854249	-0.362761583	-5.81E-23	-3.50E+01
**6**	-0.107283796	-2.919342286	-0.583868457	-9.35E-23	-5.63E+01
**CUBNUC**	-0.2309295	-6.283914996	-0.785489375	-1.26E-22	-7.58E+01
**CUNMAZ**	-0.029015353	-0.789548377	-0.263182792	-4.22E-23	-2.54E+01
**DUTRIU**	-0.001003586	-0.027308989	-0.191162923	-1.03E-22	-6.18E+01
**DUTRIU01**	-0.000245402	-0.006677724	-0.046744065	-9.35E-23	-5.63E+01
**DUTRIU02**	-0.000355113	-0.00966313	-0.067641907	-8.12E-23	-4.89E+01
**EROHUP**	-0.060858333	-1.656040452	-0.184004495	-2.95E-23	-1.78E+01
**KERDUH**	-0.152014714	-4.136533196	-0.590933314	-9.47E-23	-5.70E+01
**IPEXEH**	-0.289101	-7.866842951	-0.655570246	-1.05E-22	-6.33E+01
**LELQUQ**	-0.205798125	-5.600055099	-0.700006887	-1.12E-22	-6.75E+01
**LELRAX**	-0.141651667	-3.854540162	-0.64242336	-1.03E-22	-6.20E+01
**MEQLIE**	-0.025709333	-0.699586953	-0.233195651	-3.74E-23	-2.25E+01
**POMLUA**	-0.122072	-3.321750021	-0.474535717	-7.60E-23	-4.58E+01
**VOQFIS**	-0.085835167	-2.335695054	-0.389282509	-6.24E-23	-3.76E+01
**XEVROF**	-0.000248663	-0.00676646	-0.04736522	-8.42E-23	-5.07E+01
**XIMPAL**	-0.18226825	-4.959774258	-0.619971782	-9.93E-23	-5.98E+01

KEY: LE = Lattice Energy

Table S3. Molecular pair interaction energies partitioned into Coulombic (E_{Coul}), polarization (E_{Pol}), dispersion (E_{Disp}), repulsion (E_{rep}) and total energy (E_{Tot}) contributions (kJ/mol) for **1**–**6**.

Motif	D.C. †/(Å)	E_{Coul}	E_{Pol}	E_{Disp}	E_{Rep}	E_{Tot}	Symmetry	Important Interactions
				**1**				
1	12.99	-2.9	-1.0	-9.1	7.5	-7.0	x, -1+y, z	C23–H23···O1=C1
2	8.50	-3.2	-0.5	-18.7	10.6	-13.4	1/2+x, 1/2-y, z	C25–H25···O2–C2
3	7.96	-11.0	-3.2	-20.9	15.6	-22.6	3/2-x, -1/2+y, 1/2+z	C2–H2B···O1=C1
4	4.67	-13.1	-2.5	-47.1	31.9	-37.0	1-x, 1-y, -1/2+z	C12–H12···O2–C2; C26–H26···π
5	8.86	-9.2	-3.1	-12.7	11.9	-15.8	-1/2+x, 3/2-y, z	C2–H2A···O1=C1; C12–H12···O1=C1
				**2**				
1	4.56	-7.6	-3.8	-69.1	39.5	-46.7	x, 1+y, z	C3–H3A···O3–C3; C3–H3A···π
2	11.53	-3.7	-0.5	-8.2	6.6	-7.3	1-x, -y, 1-z	H15···H15
3	9.90	-3.4	-0.9	-15.8	10.6	-11.4	x, 1/2-y, 1/2+z	C14–H14···π
4	7.28	-10.3	-5.7	-21.3	16.0	-23.7	1-x, 1/2+y, 1/2-z	C1–H1···O1=C1; C2–H2A···O1=C1
5	10.12	-2.5	-0.7	-13.8	10.2	-8.9	x, 3/2-y, 1/2+z	C23–H23···π
				**3**				
1	7.67	-10.9	-3.2	-17.7	18.5	-17.9	3/2-x, 1/2+y, 1/2-z	C15–H15···O1=C1; C2–H2B···Br1
2	8.54	-13.5	-1.7	-32.2	32.9	-23.3	-1/2+x, 1/2-y, 1/2+z	π···π
3	7.91	-8.5	-2.2	-18.4	15.0	-17.4	1/2-x, 1/2+y, 1/2-z	C25–H25···Br1; C2–H2B···O1=C1
4	10.07	-16.0	-4.9	-21.9	26.8	-23.0	-x, 1-y, 1-z	C23–H23···O1=C1; C24–H24···O1=C1
5	14.01	-1.4	-0.2	-5.6	4.2	-4.0	-3/2+x, 1/2-y, 1/2+z	C24–H24···Br1
				**4**				
1	9.81	-2.6	-0.3	-7.1	6.4	-5.3	3/2+x, 1/2-y, 1/2+z	C25–H25···Cl1; C26–H26···Cl1
2	9.79	-4.9	-0.6	-10.4	8.2	-9.6	1/2-x, -1/2+y, 3/2-z	C25–H25···Cl1; C26–H26···Cl1
3	4.92	-3.9	-2.3	-52.5	27.9	-34.3	1+x, y, z	lone pair···π
4	8.42	-9.8	-2.5	-6.5	5.1	-14.7	-1/2+x, 1/2-y, 1/2+z	C15–H15···O1=C1; C16–H16···O1=C1
5	7.70	-5.0	-2.1	-12.5	7.5	-13.1	1/2+x, 1/2-y, 1/2+z	C2–H2A···O1=C1
6	6.56	-6.0	-1.1	-38.8	25.2	-25.4	1-x, 1-y, 1-z	lone pair···π ††
7	9.04	-11.5	-2.3	-27.9	24.5	-23.1	2-x, 1-y, 1-z	C24–H24···O1=C1 ††
				**5**				
1	9.79	-7.6	-0.2	-8.5	18.2	-4.4	-x, 1/2+y, 3/2-z	Br1···Br2
2	4.10	-23.0	-2.7	-75.2	68.3	-49.6	1+x, y, z	C2–H2B···O2–C2
3	10.23	-5.9	-0.6	-14.3	13.4	-10.9	1/2-x, 1-y, 1/2+z	C13–H13···π
4	9.34	-4.5	-0.3	-12.1	10.7	-8.9	1-x, 1/2+y, 3/2-z	C26–H26···Br2
5	7.94	-20.2	-4.5	-18.0	21.2	-27.2	1/2+x, 3/2-y, 1-z	C2–H2A···O1=C1; lone pair···lone pair
6	10.61	-5.1	-0.6	-15.6	14.0	-10.8	3/2-x, 1-y, 1/2+z	C23–H23···Br1; C24–H24···Br2
				**6**				
1	4.70	-15.4	-3.4	-47.6	32.1	-40.4	-x, 1-y, 1-z	C14–H14···C4≡C3 ††
2	7.88	-22.9	-5.1	-11.2	24.6	-22.6	-x, 1-y, 2-z	C4–H4···O1=C1; C3≡C4···O1=C1 ††
3	7.94	-4.8	-2.6	-12.6	8.0	-13.0	x, 1+y, z	C3–C2···O1=C1
4	7.55	-9.0	-2.4	-19.8	12.2	-21.0	1-x, -y, 1-z	C5–H5C···O2–C2 ††;C5–H5C···O3–C5 ††
5	6.88	-2.9	-1.0	-20.3	12.3	-13.9	1-x, -y, 2-z	C2–H2A···O2–C2 ††
6	4.59	-8.9	-2.2	-44.5	23.3	-35.4	1-x, 1-y, 1-z	C5–H5A···O1=C1 ††
7	8.43	-2.3	-2.6	-7.5	6.3	-7.1	1-x, 1-y, 2-z	C1–H1···O1=C1 ††

† Centroid distance; †† Duplicated interactions

NMR

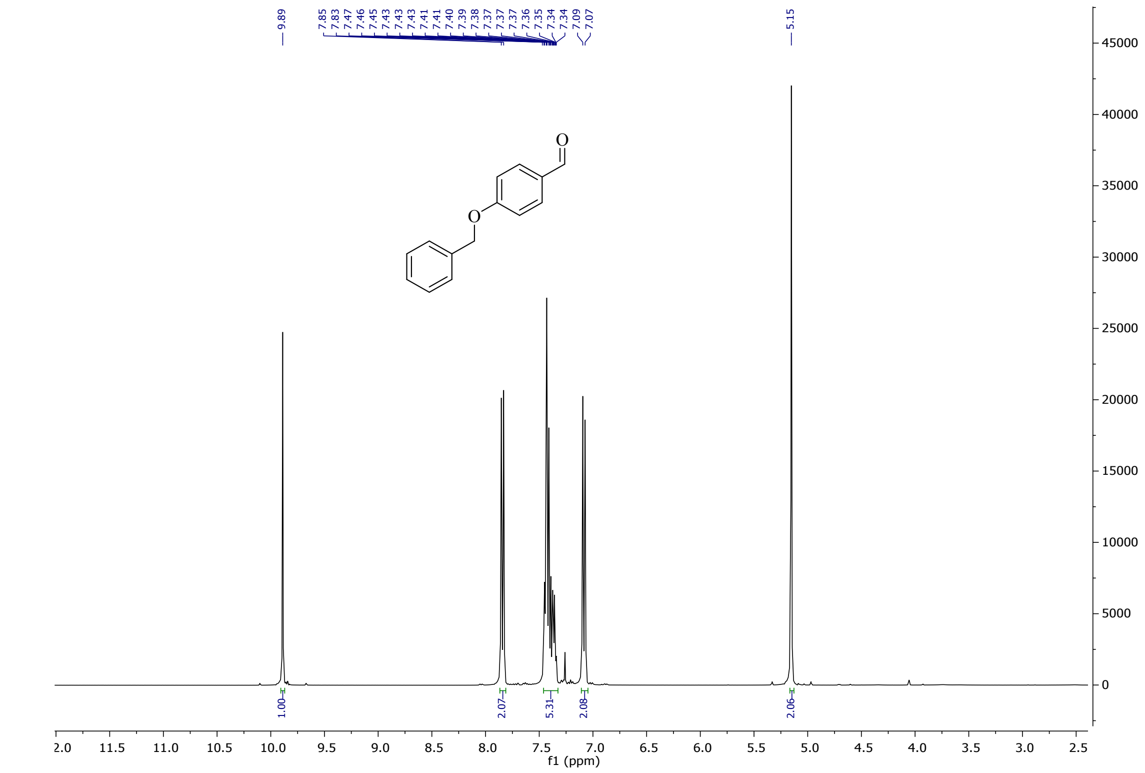

Fig.S2a 400 MHz ^{1}H NMR spectrum of compound **1** in $CDCl_3$

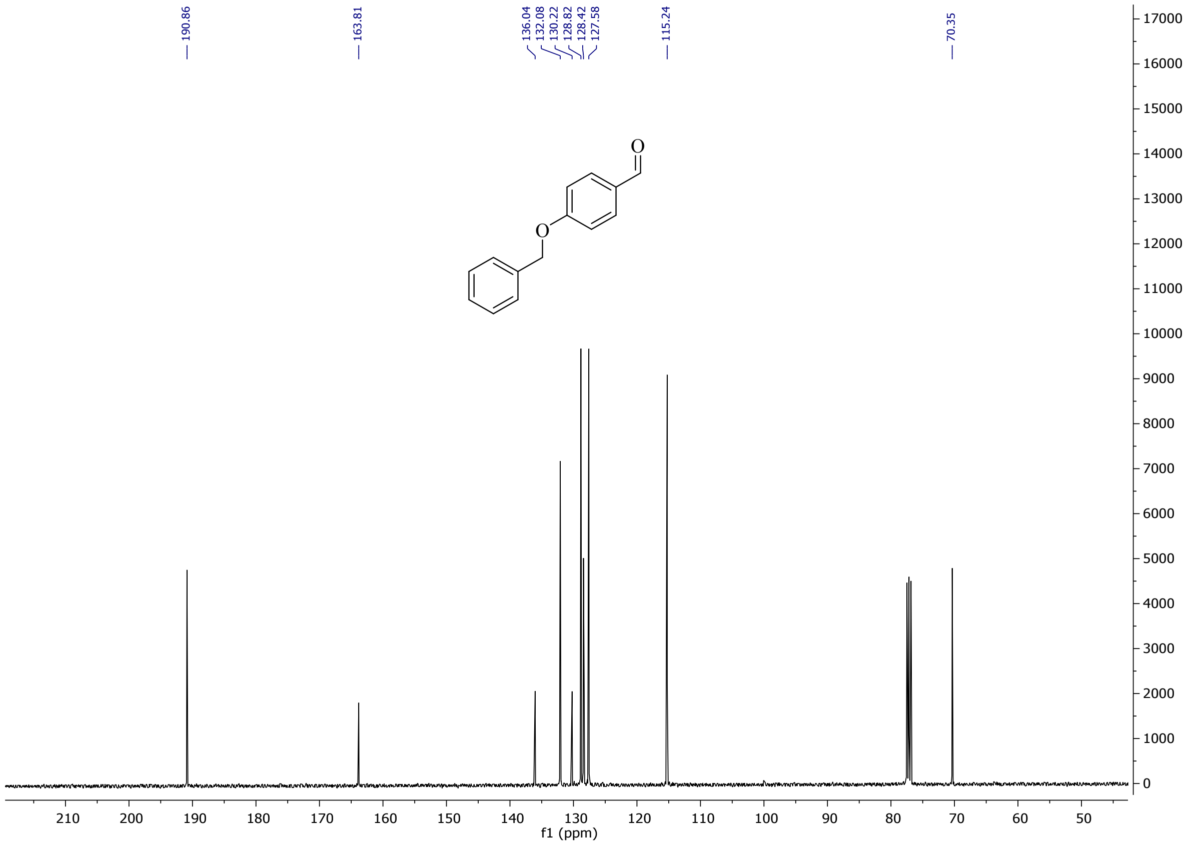

Fig. S2b 100 MHz ^{13}C NMR spectrum of compound **1** in $CDCl_3$.

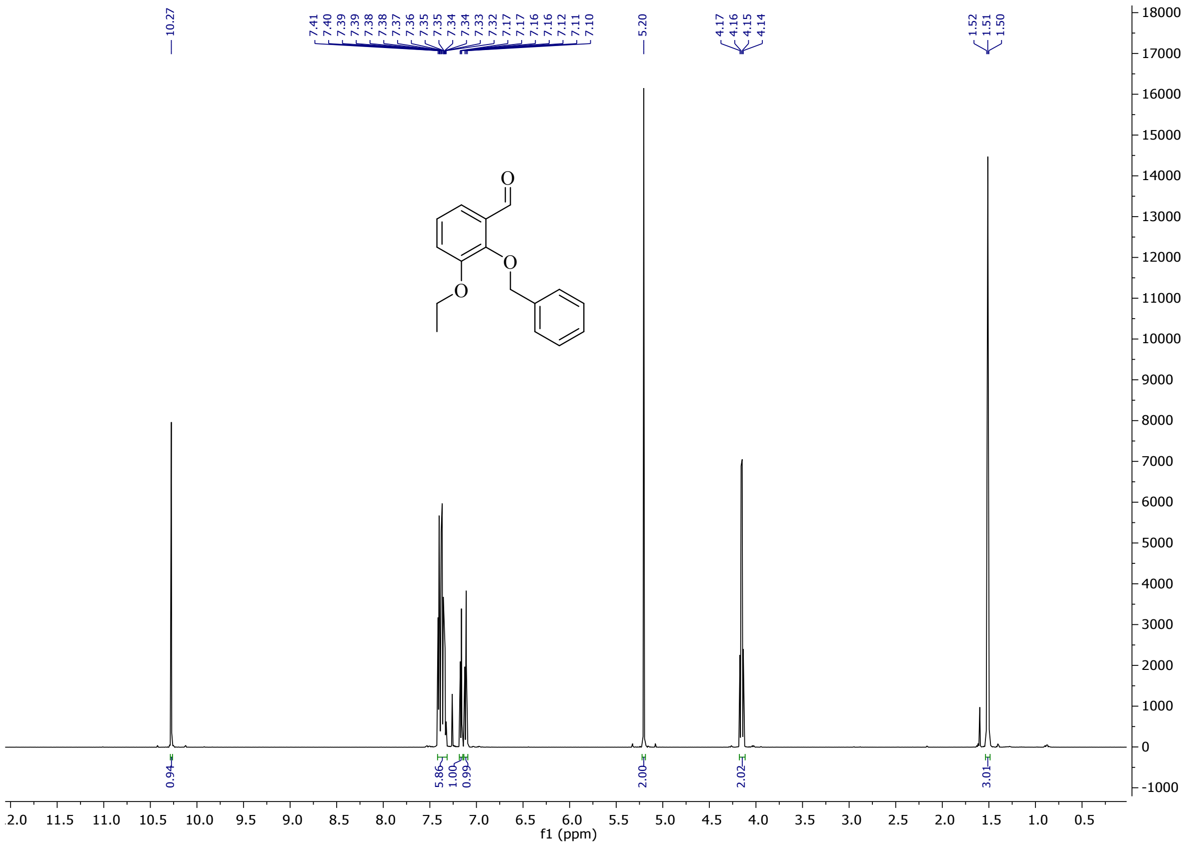

Fig.S3a 600 MHz ^{1}H NMR spectrum of compound **2** in $CDCl_3$.

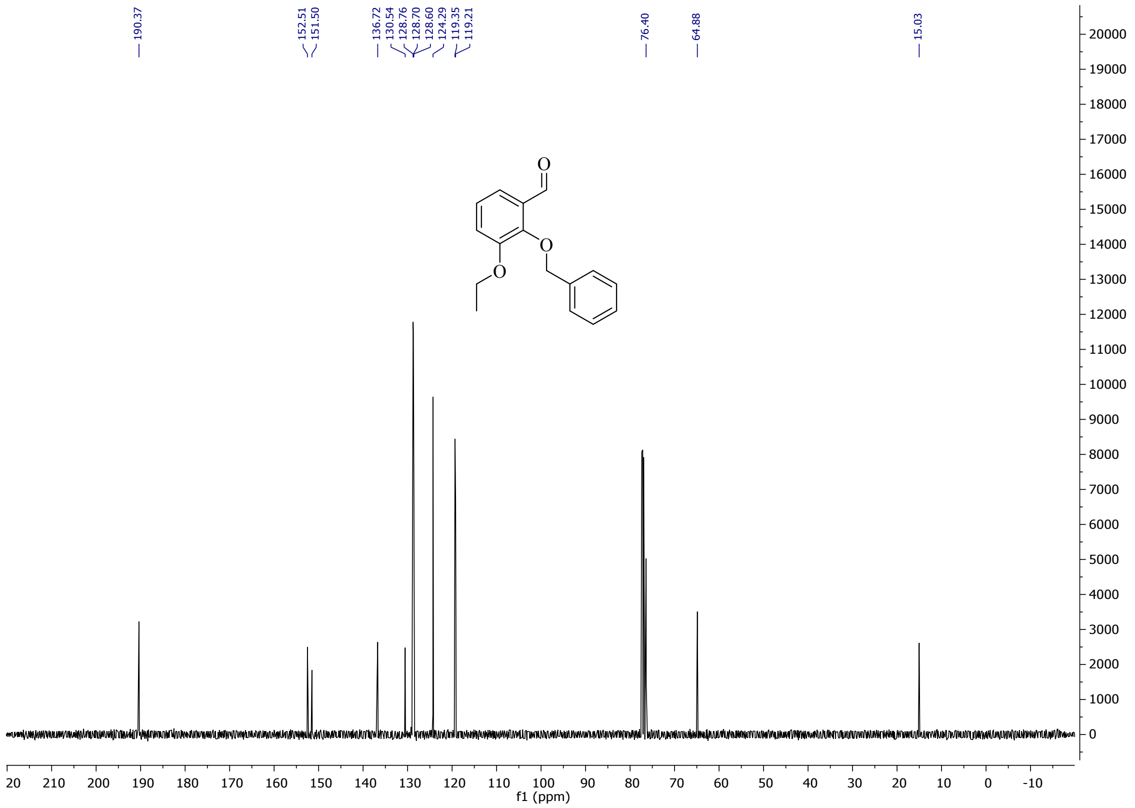

Fig. S3b 150 MHz ^{13}C NMR spectrum of compound **2** in $CDCl_3$.

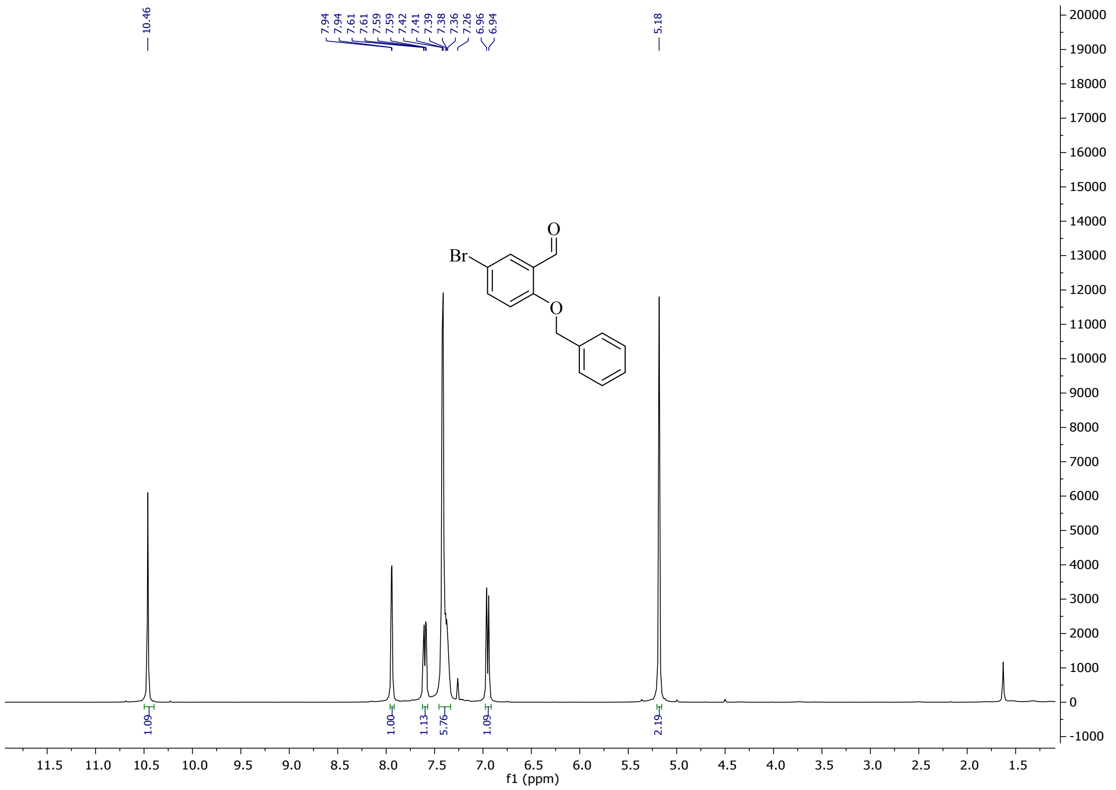

Fig.S4a 400 MHz ^{1}H NMR spectrum of compound **3** in $CDCl_3$.

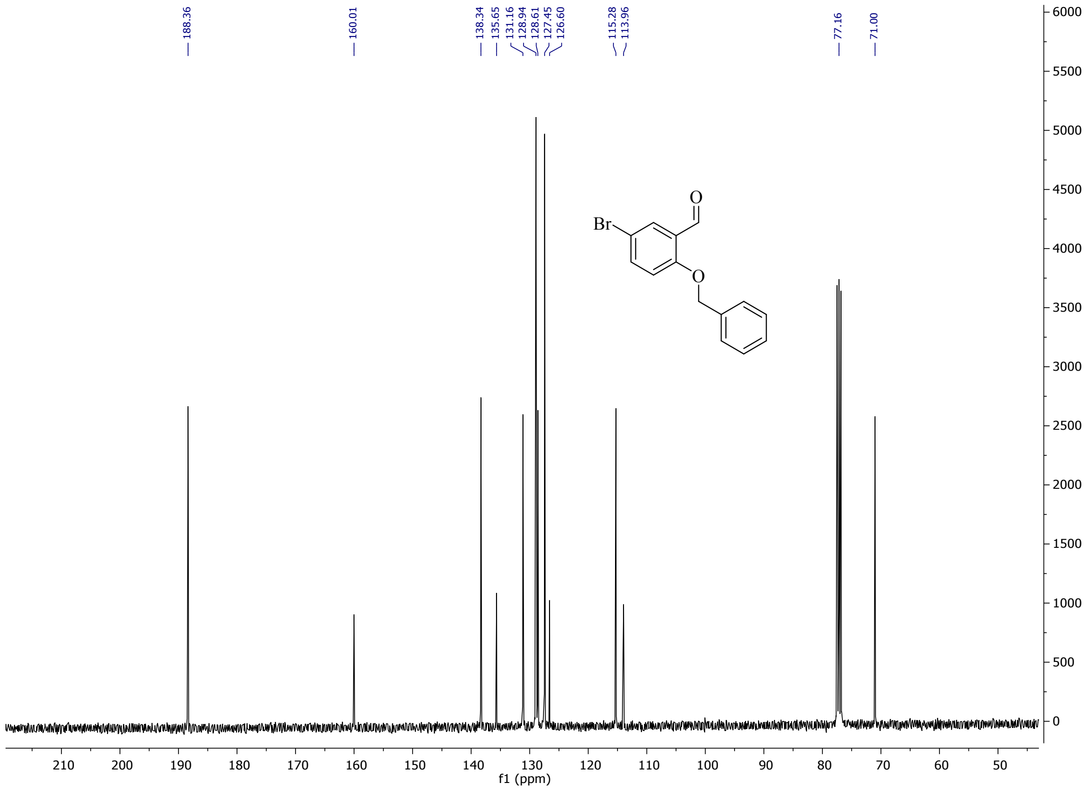

Fig.S4b 100 MHz ^{13}C NMR spectrum of compound **3** in $CDCl_3$.

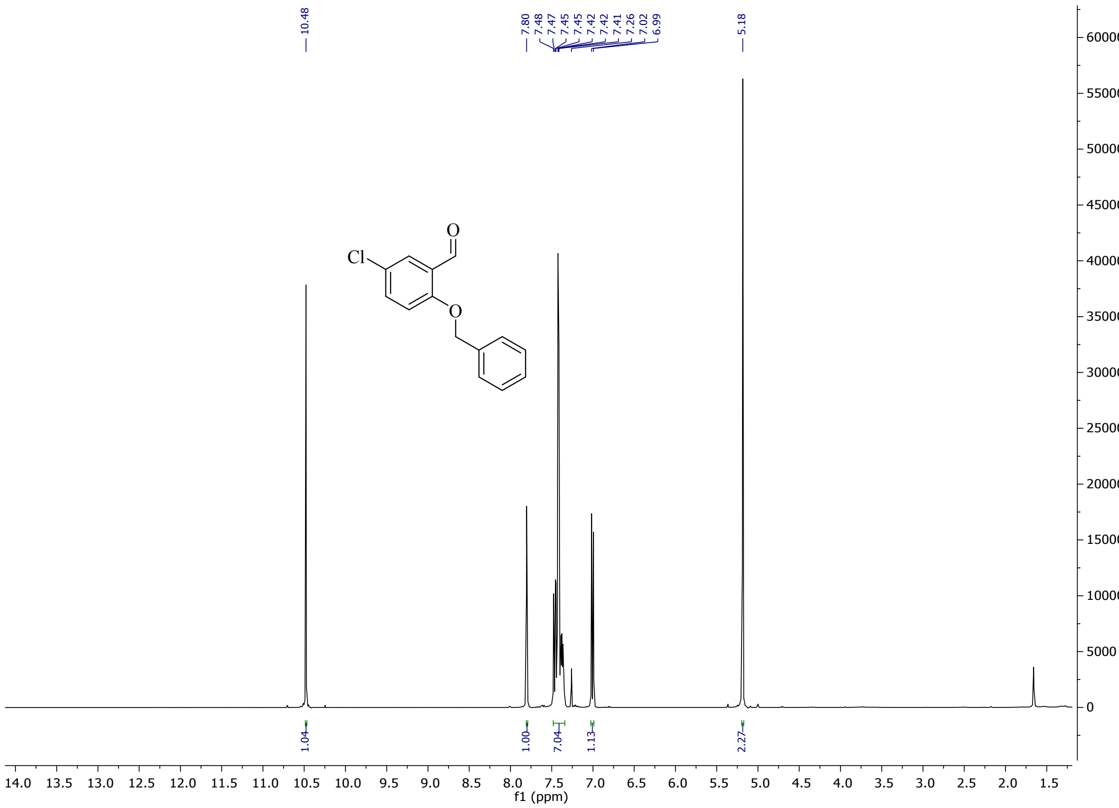

Fig.S5a 400 MHz ^{1}H NMR spectrum of compound **4** in $CDCl_3$.

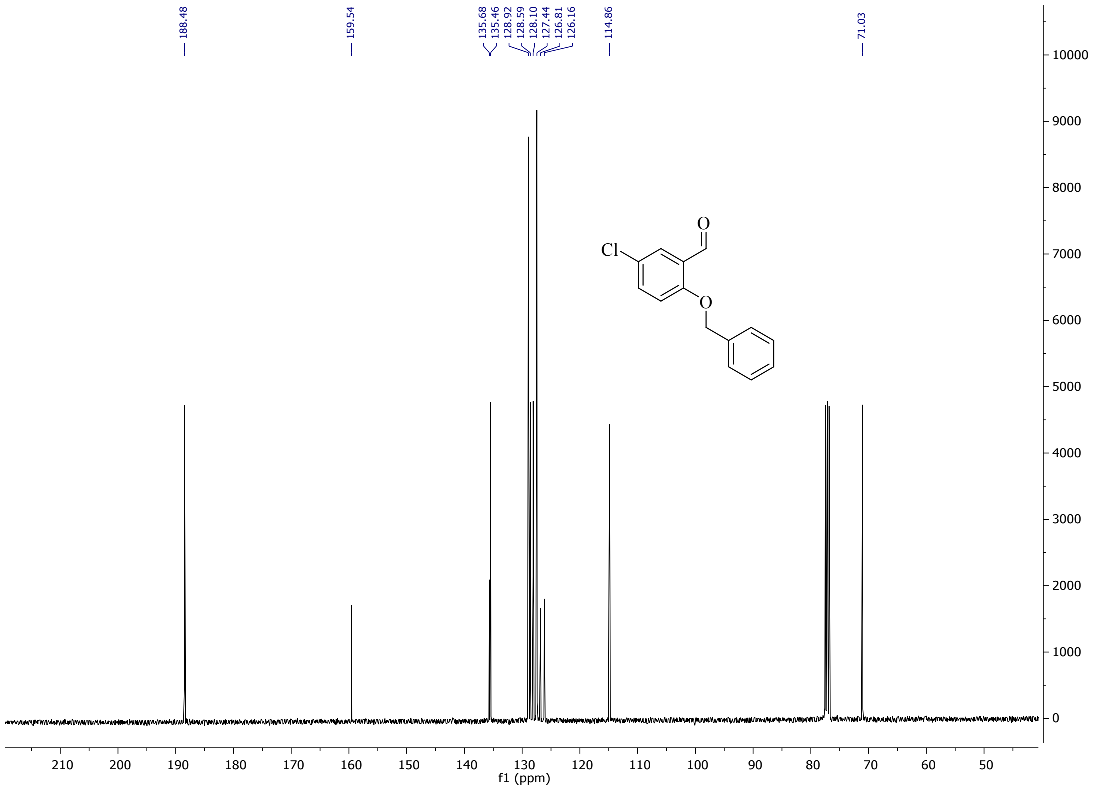

Fig.S5b 100 MHz ^{13}C NMR spectrum of compound **4** in $CDCl_3$.

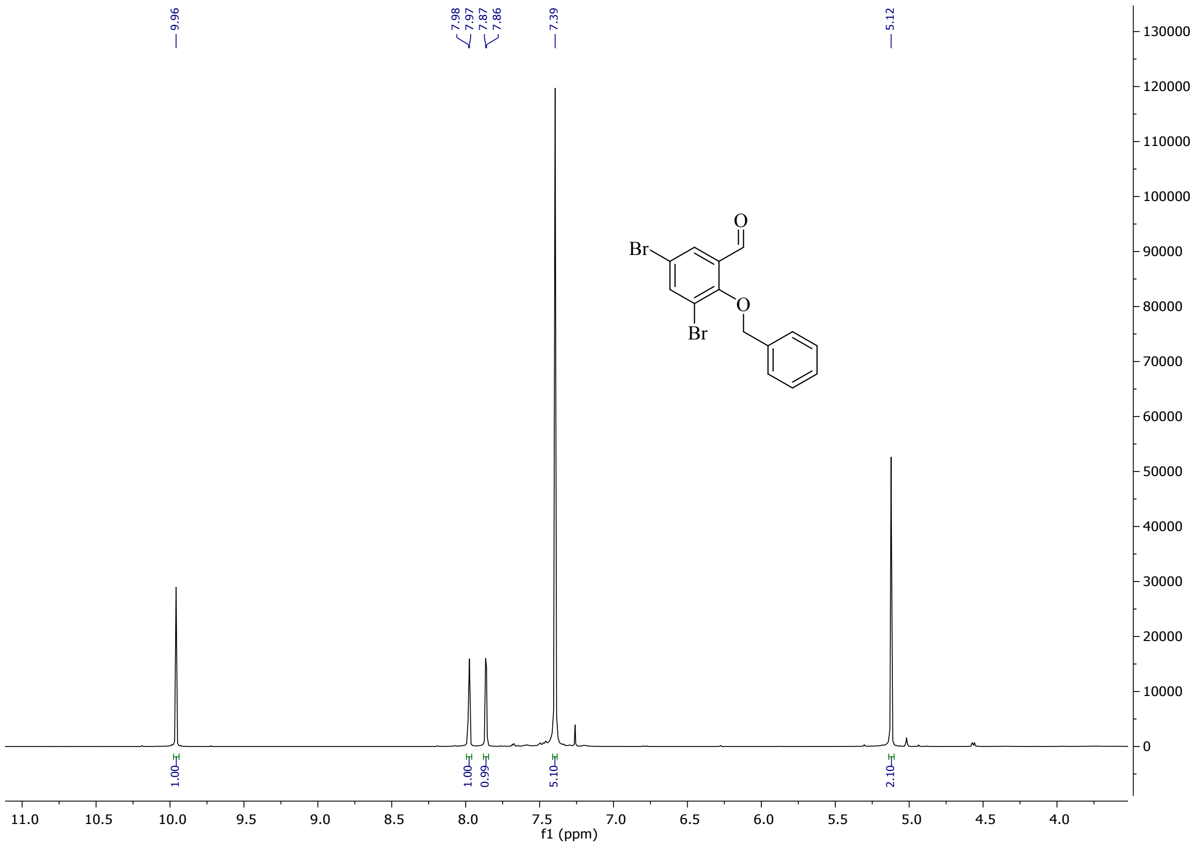

Fig.S6a 400 MHz ^{1}H NMR spectrum of compound **5** in $CDCl_3$.

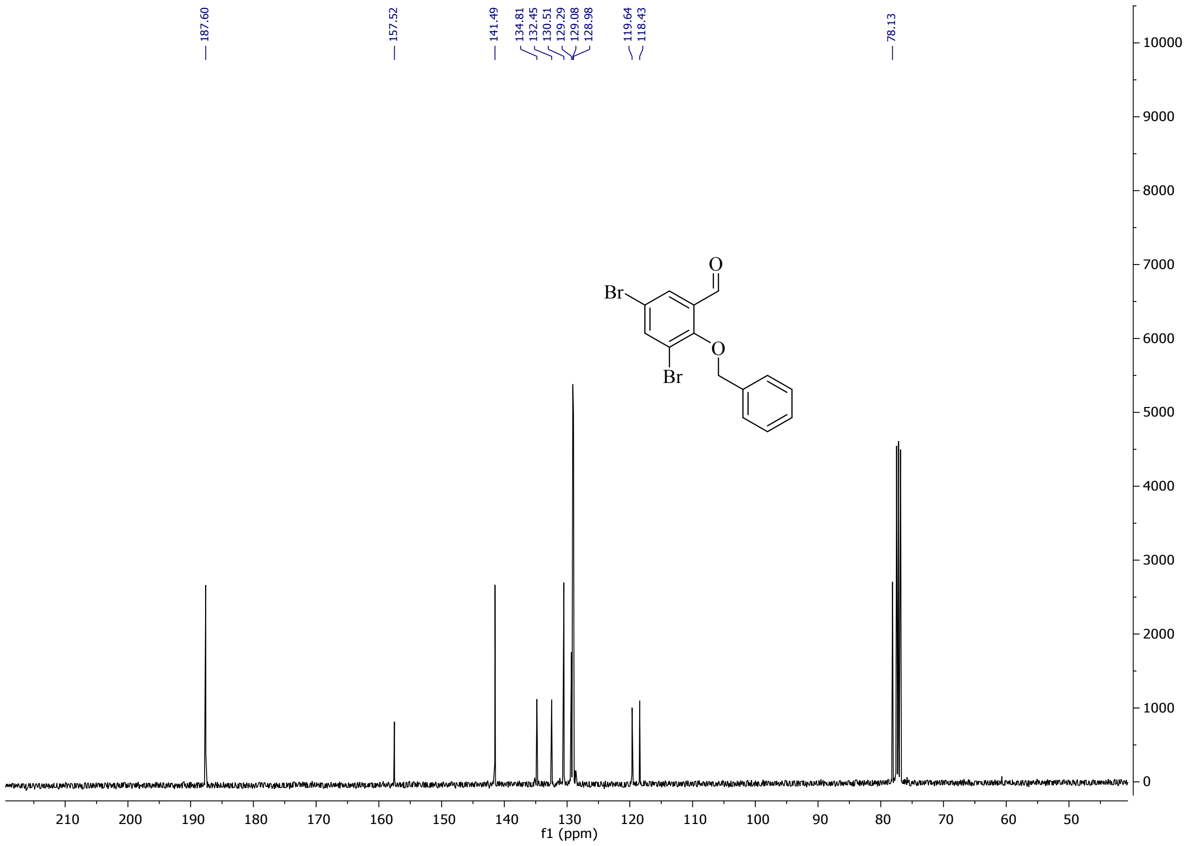

Fig.S6b 100 MHz ^{13}C NMR spectrum of compound **5** in $CDCl_3$.

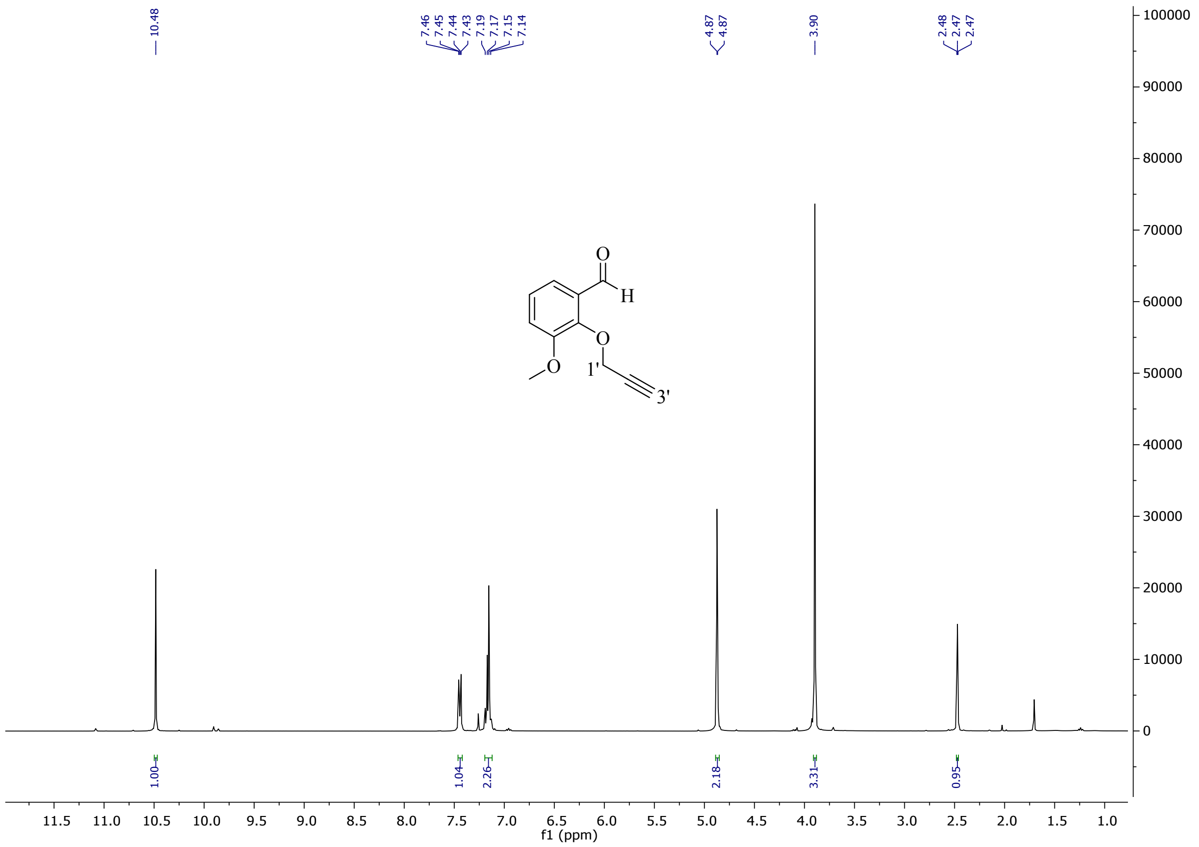

Fig.S7a 400 MHz ^1H NMR spectrum of compound **6** in $CDCl_3$

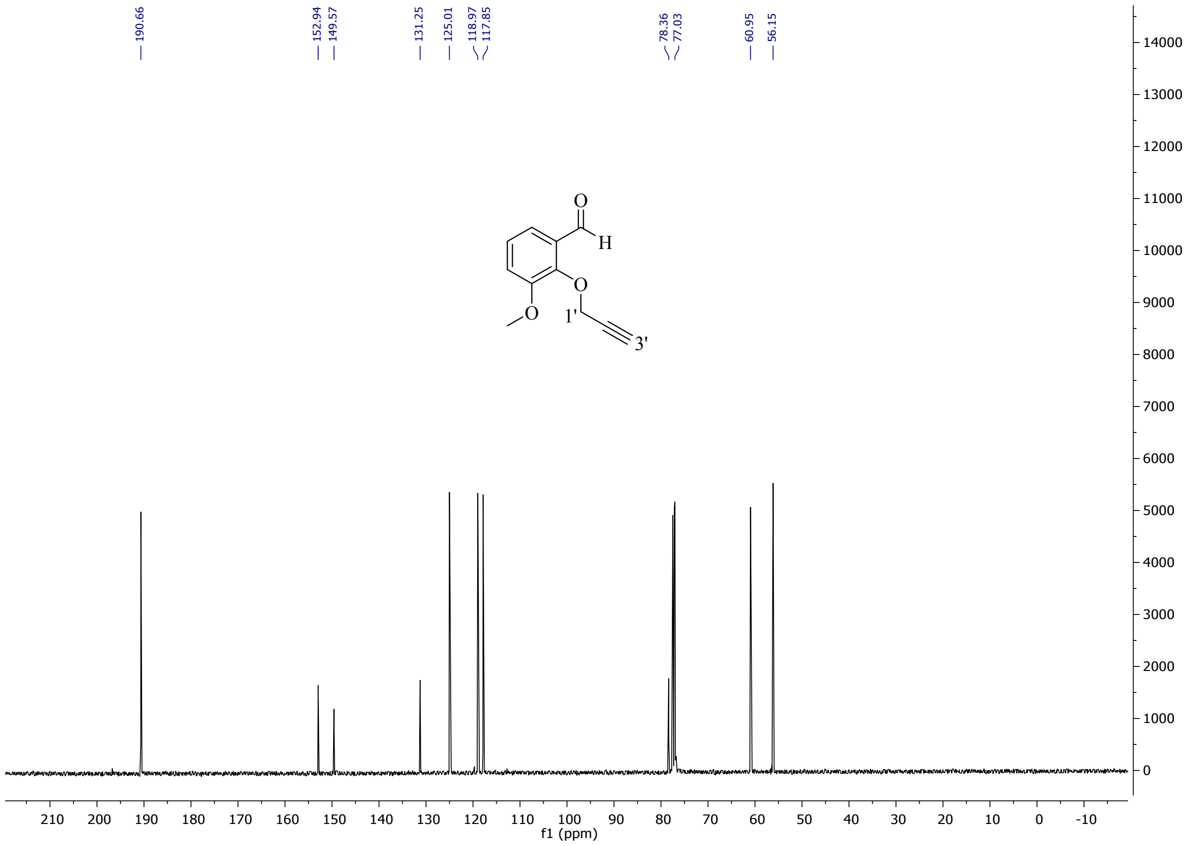

Fig.S7b 100 MHz ^{13}C NMR spectrum of compound **6** in $CDCl_3$.

This journal is © The Royal Society of Chemistry 2019

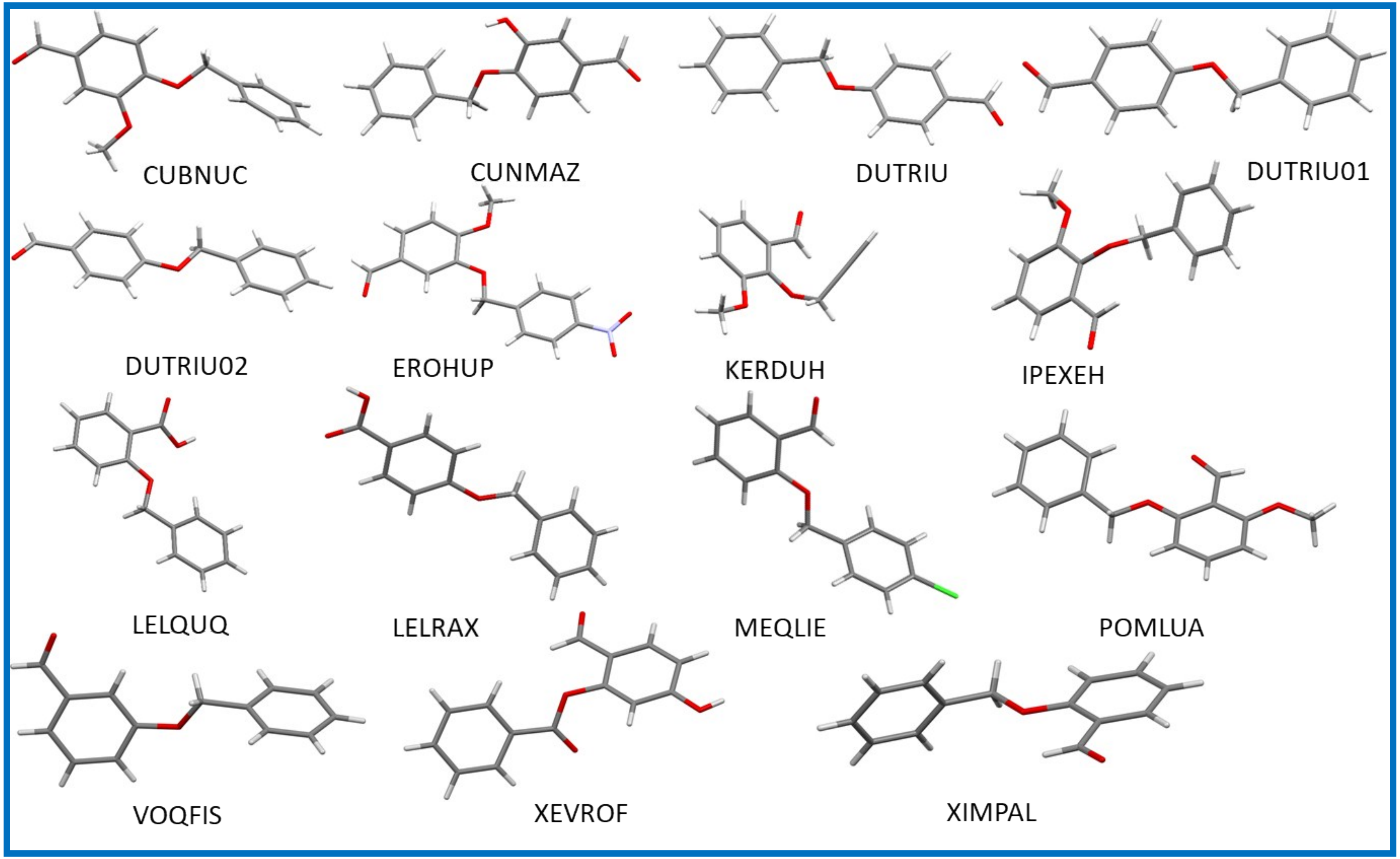

Fig. S8 Benzyloxybenzaldehydes than their analogous structures.
